# Ender3 3D printer kit transformed into open, programmable syringe pump set

**DOI:** 10.1016/j.ohx.2021.e00219

**Published:** 2021-08-03

**Authors:** Sander Baas, Vittorio Saggiomo

**Affiliations:** Laboratory of BioNanoTechnology, Bornse Weilanden 9, Wageningen University and Research, Wageningen, The Netherlands

**Keywords:** Syringe pumps, Flow chemistry, Microfluidics

## Abstract

A cheap, open source 3D printer (Creality Ender 3) is transformed into an Open Hardware, programmable syringe pump set. Only 3 parts need to be purchased outside of the printer kit. All other parts are either in the Ender 3 kit, or can be 3D printed. No prior knowledge in electronics or programming languages is required. The pumps are controlled by the 3D printer firmware and motherboard and programmed in simple G-code text files. The total cost of a three pumps setup is ∼€170. The pumps are capable of reaching stable flows down to 5 µL/min using cheap, disposable 10 mL syringes. Higher flow speeds are also achievable, in the order of mL/min.

## Graphical abstract

**Figure:**
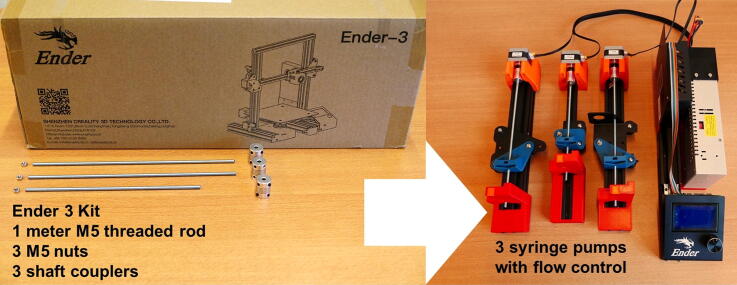

**Specifications Table:** 

Hardware name	Please specify the name of the hardware that you invented/customized
Subject area	• Chemistry and Biochemistry • Biological Sciences (e.g. Microbiology and Biochemistry) • Educational Tools and Open Source Alternatives to Existing Infrastructure • General
Hardware type	• Biological sample handling and preparation • Mechanical engineering and materials science • Syringe Pumps
Open Source License	CC-BY 4.0
Cost of Hardware	€170
Source File Repository	A live version of the repository can be find on github: https://github.com/Vsaggiomo/Ender3-syringe-pumps Zenodo: https://zenodo.org/record/5139558

## Hardware in context

Syringe pumps are an excellent tool used in many different fields: from physics [1], flow chemistry [2], microfluidics [3], [4], to biology and microscopy [5]. However, even a simple syringe pump, with little possibility of automation, can cost close to €1000 [6]. Because of the high cost and the limited automation capability, many groups around the world have switched to a Do It Yourself (DIY) approach [7], [8]. Many DIY pumps have been published in the last few years using stepper motors and lead screws to translate the rotational motion of the motor to linear motion of a syringe plunger. Recently the group of Pachter published a Python controllable pumps system, called Poseidon [9], where the whole pump frame is 3D printed. Some designs use ready-made parts for the frame, such as metal rods [10] or aluminum extrusions [11]. Henriques’ group built a set of pumps using Lego blocks and controlled them using Arduino [12]. In general, Arduino is a popular control board for DIY syringe pump systems [6], [13], [14], [15]. The Raspberry Pi single board computer is also used for pump control [16], also in combination with commercial pumps [17]. Non syringe-based DIY pump designs, such as peristaltic [18], [19], [20] and manually operated pumps [21], [22] have also been demonstrated. Open source (syringe) pumps have found many applications, for example in hydrogel printing and bioprinting [23], [24], in vivo imaging[25], printing of TLC layers [26] and blood plasma separation [27].

Although all these pumps work correctly and are an excellent opportunity to enter the Open Hardware DIY instruments field, building and programming them requires, first, the sourcing of all the parts from different vendors and then, some previous knowledge in programming to use them. The sourcing of the parts may result in a scavenger hunt, with parts changing name or vendor and specs over time. Another barrier is the knowledge of electronics and programming that is usually required to build and use these pumps. These two problems may hinder the widespread adoption of DIY pumps among researchers without previous knowledge in these areas. Moreover, they may not be willing to spend time in a build process if they first must acquire these skills.

To solve the issues of acquiring many parts and going through a complicated build process, we leverage the use of a 3D printer electronics and mechanics for building and controlling a set of three syringe pumps. A standard fused deposition modelling (FDM) 3D printer has, in fact, all the components required to build a set of syringe pumps: stepper motors, motherboard, Power Supply Unit (PSU), and linear motion systems in the three XYZ axes. Moreover, the program used for controlling 3D printers, usually Marlin [28] working with G-code files, can be exploited for controlling the syringe pumps. Marlin is widespread in Open Hardware projects for motion control. It has been used for sampling [29], [30], [31], positioning in neurophysiology research [32] as well as automated planar chromatography [33]. Using Marlin in this project avoids the need of programming the pumps from scratch, using microcontrollers like Arduino or the Linux based Raspberry Pi.

**Fig. 1:**
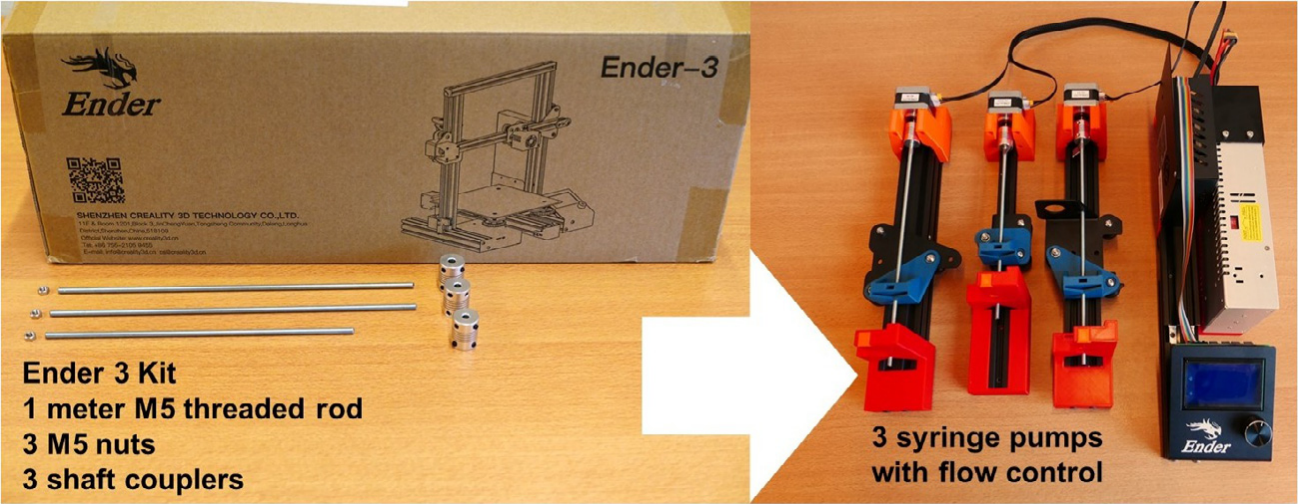
An Ender 3 3D printer kit can be repurposed to build a three channel syringe pump system. Additional purchased components are limited to one meter M5 threaded rod, three M5 nuts and three 5 × 5 shaft couplers. All custom parts can be 3D printed.

In this paper, we show how to build and use a set of 3D pumps by repurposing a Creality Ender3 [34], one of the cheapest FDM printers on the market. Apart from three shaft couplers, one M5 threaded rod and three M5 nuts, everything needed for this build is included in the Ender 3 box, even the screws and the tools needed for assembly. Some additional parts that are needed for the syringe and motors connections are 3D printed, meaning that the Ender 3 can be first built for printing the parts, and then disassembled for building the syringe pumps (Fig. 1). We also show how to use G-code for controlling and automating the pump system.

This approach of repurposing a 3D printer and control via G-code for use as syringe pumps is cheap, fast, and simple [35]. It opens the opportunity of acquiring and using syringe pumps in laboratories around the world, independent of funding and previous knowledge in programming and electronics.

## Hardware description

This project describes the procedure to repurpose a 3D Printer to a set of three syringe pumps. All the components, even the screws and the tools needed for the build are in the 3D printer box, simplifying the acquisition of each separate component. The only additional parts to be purchased are 5 × 5 shaft couplers, M5 nuts and M5 threaded rods, available at any hardware store. The 3D printed parts are supplied in their original CAD format (.F3D) as well as .STL files ready to be 3D printed. Printable parts were designed using Fusion 360 from Autodesk. For this specific project, we used a Creality Ender 3 3D printer, as it is one of the cheapest FDM on the market, and it is open source/open hardware [36]. However, as all FDM 3D printers work by moving a XYZ stage, this method can be applied to any 3D printer by redesigning the 3D printed parts, while the G-code programming will remain the same.

In fact, the programming of the pumps is also easy as it leverages the Marlin firmware already present on many 3D printer motherboards, and G-code files for controlling the pumps can be easily written as text files. This means that even when the Creality Ender 3 becomes discontinued, the same conversion approach can be used for other i3 style 3D printers. This would require a redesign of the 3D printed parts, but the programming, control and mechanical principles remain the same. The same design here presented has already worked on the Ender 3 Pro, and the Ender 3 V2 with a small modification of the PSU 3D printed unit, now present in the GitHub. Moreover, the parts are off the shelf components (stepper motors, aluminum rails, control board). This means that in case of unavailability of the Ender 3, parts can be sourced separately, and the pumps can still be built. This may not be as cost effective or user friendly compared to the current work, but it does not render this setup completely dependent on Ender 3 availability.

This project comprises of an easy to assemble and program syringe pump set, which can be used for:

• Flow chemistry
• Microfluidics
• Biology and Microscopy (for example for automatic staining and fixing)

Moreover, this project can be used as a blueprint to expand the possibilities of repurposing 3D printers for laboratory equipment. For example, in the 3D printer kit, there is a fourth motor, two heating units with thermistor controls and three end stops, which are not used in this project, but can be useful to build other instruments. The project files and description are available on github [37].

## Design files

### Print settings

All the design files listed in Table 1 were printed using PLA material, at 0.2 mm layer height, 20% infill, with two perimeter wall thickness. The designs are printable without using support structures.

### Design files summary

Table 1

**Table 1:** Design file overview, with the file name of the part, the available file types, license type and the repository location.

Design file name	File type	Open source license	Location of the file
Clamp Bar Long v1	F3D (Fusion 360), STL	CC-BY 4.0	https://github.com/Vsaggiomo/Ender3-syringe-pumps
Clamp Bar Short v1	F3D (Fusion 360), STL	CC-BY 4.0	https://github.com/Vsaggiomo/Ender3-syringe-pumps
Clamp Bar v1	F3D (Fusion 360), STL	CC-BY 4.0	https://github.com/Vsaggiomo/Ender3-syringe-pumps
Dovetail Cap v1	F3D (Fusion 360), STL	CC-BY 4.0	https://github.com/Vsaggiomo/Ender3-syringe-pumps
E End Mount v1	F3D (Fusion 360), STL	CC-BY 4.0	https://github.com/Vsaggiomo/Ender3-syringe-pumps
E Slide Mount v1	F3D (Fusion 360), STL	CC-BY 4.0	https://github.com/Vsaggiomo/Ender3-syringe-pumps
E Stepper Mount v1	F3D (Fusion 360), STL	CC-BY 4.0	https://github.com/Vsaggiomo/Ender3-syringe-pumps
Electronics Base v1	F3D (Fusion 360), STL	CC-BY 4.0	https://github.com/Vsaggiomo/Ender3-syringe-pumps
PSU Mount v1	F3D (Fusion 360), STL	CC-BY 4.0	https://github.com/Vsaggiomo/Ender3-syringe-pumps
X Motor Slide Mount v1	F3D (Fusion 360), STL	CC-BY 4.0	https://github.com/Vsaggiomo/Ender3-syringe-pumps
X Slide Mount v1	F3D (Fusion 360), STL	CC-BY 4.0	https://github.com/Vsaggiomo/Ender3-syringe-pumps
X X Motor End Mount v1	F3D (Fusion 360), STL	CC-BY 4.0	https://github.com/Vsaggiomo/Ender3-syringe-pumps
X X Motor Stepper Mount v1	F3D (Fusion 360), STL	CC-BY 4.0	https://github.com/Vsaggiomo/Ender3-syringe-pumps
Press Fit Pulley Extractor	STL	CC-BY 4.0	https://www.thingiverse.com/thing:3593964 Designed by Jakob Schmitz And https://github.com/Vsaggiomo/Ender3-syringe-pumps

### Part denominations

Some parts are not universal to all three syringe pumps and are denoted by ‘E’, ‘X’ or ‘X Motor’. This refers to the sliding plates (with 3 roller wheels each) that are reused from the printer structure. One slide plate belongs to the extruder assembly (‘E’), one to the X axis motor mount (‘X Motor’) and one to the X axis free running support (‘X’). Some parts are shared between the X Slide Plate and X Motor Slide Plate and are denoted ‘X X Motor <part name>’.

### Clamp Bar (Long, Normal, Short)

The Clamp Bar is used to keep the syringe pressed against the End Mount frame, under pressure of a spring. Three lengths are available, in order to accommodate different syringe diameters, if the spring pressure is insufficient, or too great.

### Dovetail Cap

A cap to lock the spring and Clamp Bar in place. It slides in with a dovetail-type mechanism.

### E End Mount

The mount for the E Plate Pump in which the syringe body is clamped.

### E Slide Mount

The mount that is installed on the E Slide Plate, which moves the syringe plunger.

### E Stepper Mount

The E Stepper Mount is used to mount the stepper motor, driving the leadscrew.

### Electronics Base

A base to support the Electronics Box when it is mounted on the Control Unit.

### PSU Mount

A rectangular block that connects the PSU to the Control Unit.

### Motor Slide Mount

The mount that is installed on the X Motor Slide Plate, which moves the syringe plunger.

### Slide Mount

The mount that is installed on the X Slide Plate, which moves the syringe plunger.

### X Motor End Mount

The mount for the X Plate and X Motor Plate in which the syringe body is clamped.

### X Motor Stepper Mount

The X X Motor Stepper Mount is used to mount the stepper motor, driving the leadscrew. It is used in both the X Plate and X Motor Plate pumps.

### Press Fit Pulley Extractor

This tool is used to remove the pulleys that are press fit onto the stepper motor shafts.

## Bill of materials

As stated, this project aims to enable researchers to build an Open Hardware syringe pump system, with the need for purchasing as little externally sourced components as possible. The Bill of Materials therefore consists mainly of 3D printed parts, designed to construct this pump system. The threaded rod and M5 nuts should be readily available at a hardware store. The Ender 3 can be bought online. Some online vendors specialize in the sale of 3D printers, will also sell parts like the 5 × 5 shaft coupler. The shaft coupler should also be available on major online stores.

**Fig. 2:**
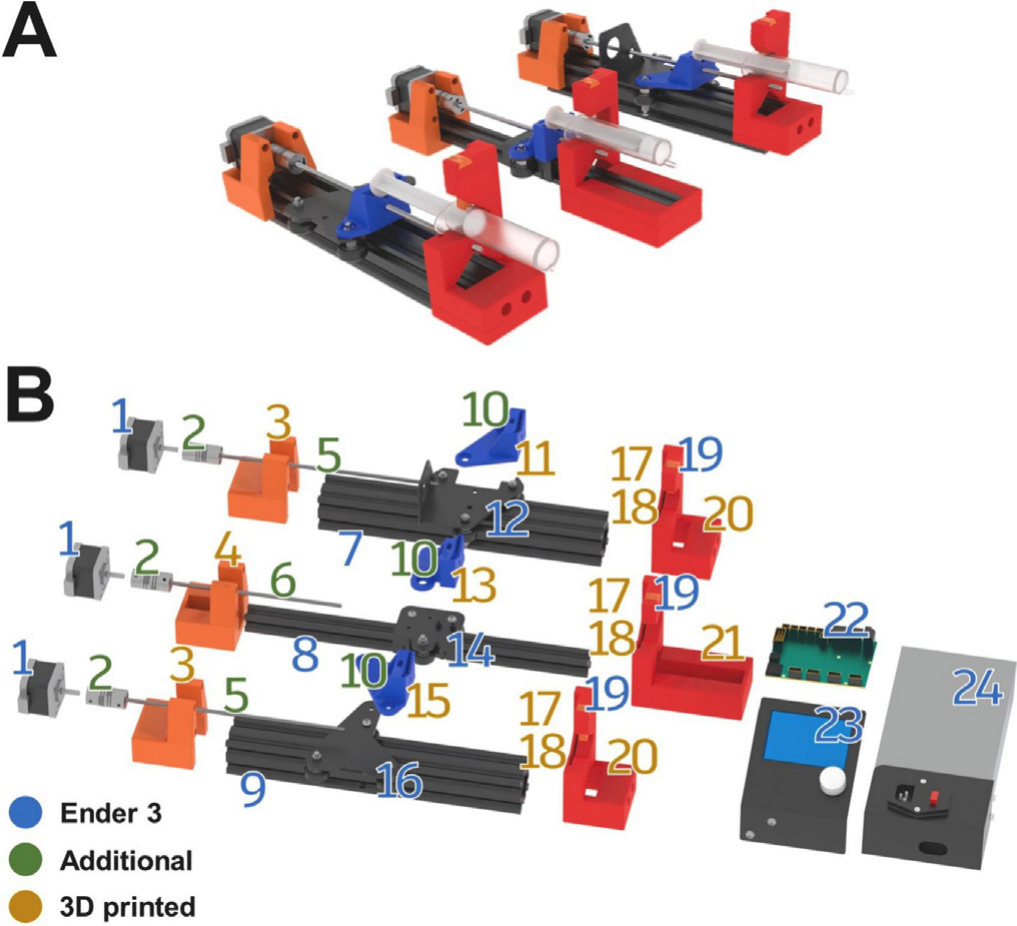
A) Render of the three pump channels. B) Exploded view of pump parts with number indication the parts (Table 3). Blue numbers: Parts included in the Ender 3 kit. Green numbers: Additional purchased parts. Yellow numbers: 3D printed designs. (For interpretation of the references to colour in this figure legend, the reader is referred to the web version of this article.)

A render of the three pump channels is shown in Fig. 2A. Also shown, is an exploded view of the system, with all parts numbered (Fig. 2B). An overview of these parts can be found in Table 2, Table 3, below. This gives the builder a general indication of which parts are used and where. A detailed account of the construction process can be found in the build instructions in the next section.

**Table 2:** The Bill of Materials, listing all the parts that need to be purchased, the amount, cost, vendor and material type.

Component	Number	Cost per unit – €	Total cost – €	Source of materials	Material type
M5 threaded rod (1 m)	1	3.79	3.79	Hardware store	Stainless steel
M5 nut	3	0.54	3.59 (per 20)	Hardware store	Stainless steel
5 × 5 shaft coupler	3	3.68	11.04	123-3D.nl	Aluminium
Ender 3 Kit	1	140	140	Aliexpress	DIY 3D printer kit
3D printed parts (500 g)	1 kg roll of PLA filament	20	10	123-3D.nl	PLA

**Table 3:** Overview of the parts in the exploded view (Fig. 2B). 3D printed parts, commercially bought parts and (sub)assemblies from the Ender 3 are included.

Number	Part	Source
1	Stepper Motor	Ender 3
2	5 × 5 Shaft Coupler	Additional
3	X X Motor Stepper Mount	3D Printed
4	E Stepper Mount	3D Printed
5	27 cm M5 leadscrew	Additional
6	22 cm M5 leadscrew	Additional
7	40 × 40 rail	Ender 3
8	20 × 20 rail	Ender 3
9	40 × 40 rail	Ender 3
10	M5 nut	Additional
11	X X Motor Slide Mount	3D Printed
12	X Motor Plate	Ender 3
13	E Slide Mount	3D Printed
14	E Plate	Ender 3
15	X Slide Mount	3D Printed
16	X Plate	Ender 3
17	Dovetail Cap	3D Printed
18	Clamp Bar	3D Printed
19	Spring	Ender 3
20	X X Motor End Mount	3D Printed
21	E End Mount	3D Printed
22	Control Board	Ender 3
23	LCD Screen	Ender 3
24	PSU	Ender 3

## Build instructions

In the following section, the build process of the Ender 3 based pump system will be explained. Assembly instructions, as well as indications of how to disassemble and reuse existing parts will be given. The guide consists of 37 steps and will result in three pump channels, as well as a Control Unit. Before starting the assembly process, make sure to print the pulley removal tool (https://www.thingiverse.com/thing:3593964) that is used in step 18. Without this tool, the build process cannot be completed. See step 18 for further instructions.

**Step 1: Layout**

**Fig. 3:**
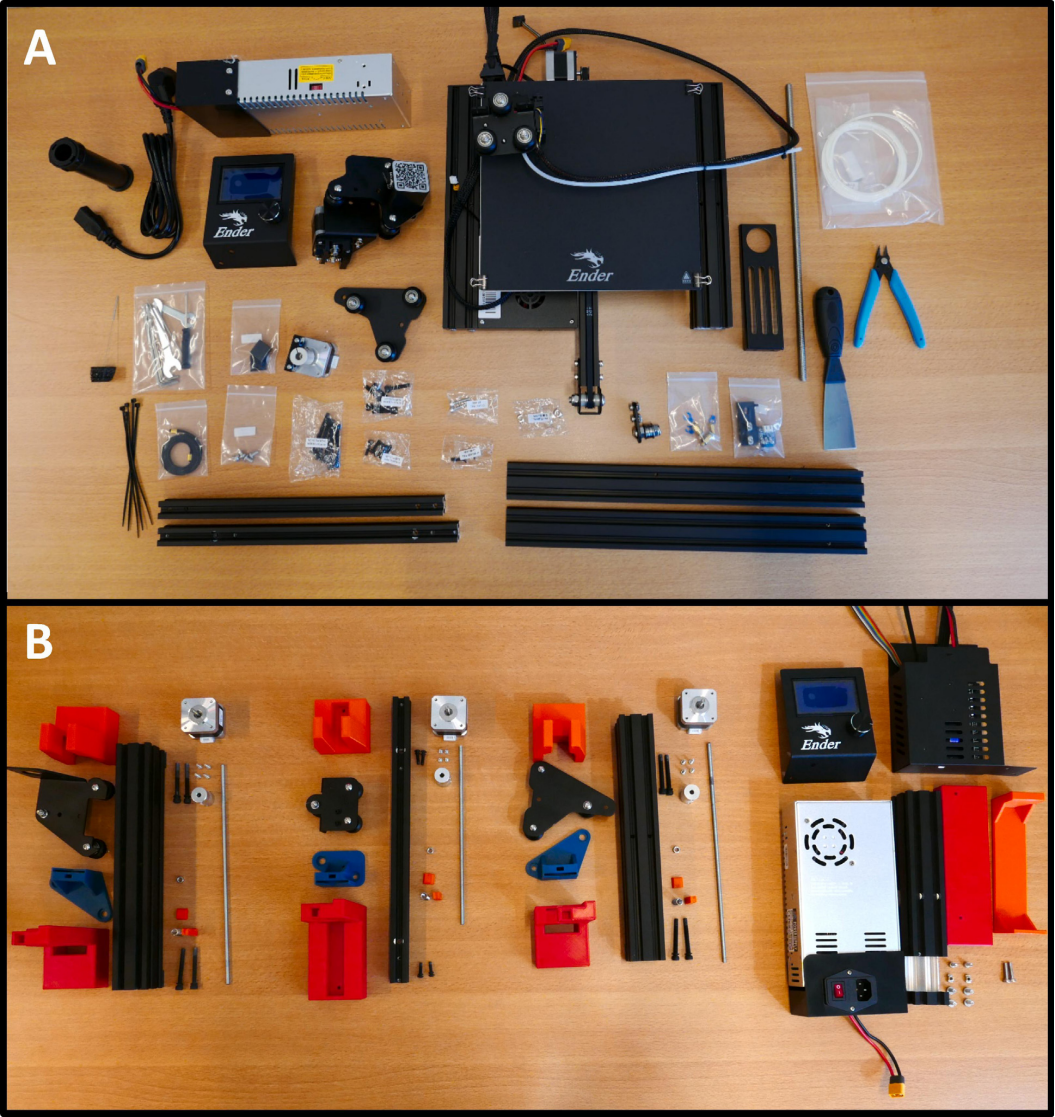
A) All parts included in the Ender 3 kit. B) All parts used in the syringe pump system.

Start with laying out all the parts and part bags included in the kit (Fig. 3A). All parts that used in the syringe pump system are shown Fig. 3B. Fig. 3B only serves as a general reference for the build process. Some of the parts shown will be extracted from the kit during the build steps, others are 3D printed.

**Step 2: Collect the necessary parts for the E Plate Pump frame**

**Fig. 4:**
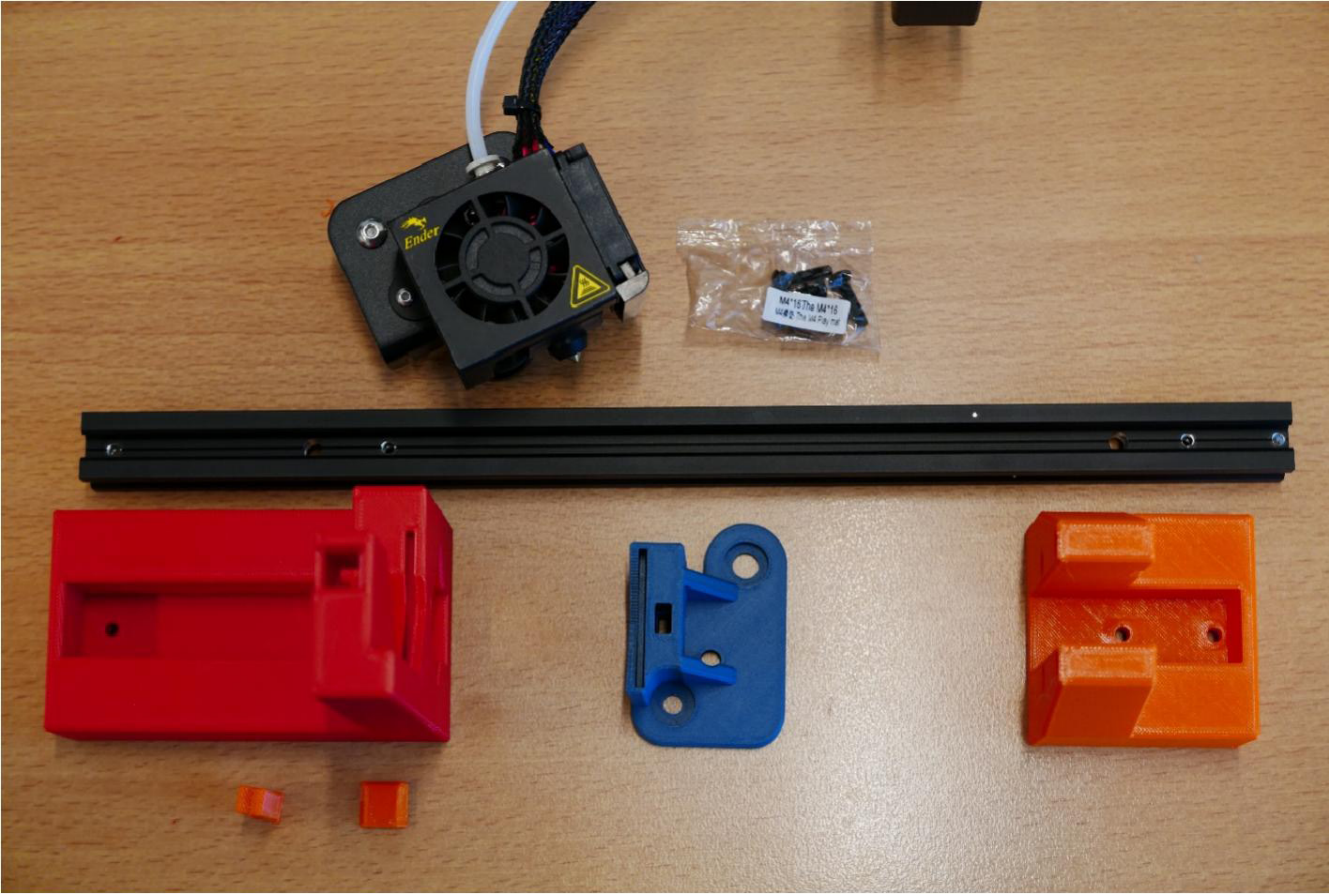
Necessary parts to build the Extruder Plate Pump Channel frame: Extruder head, M4 × 16 screws, 20 × 20 rail, E End Mount, E Slide Mount, E Stepper Mount, Clamp Bar and Dovetail Cap.

To build the Extruder Plate Pump frame, take the extruder head, the long 20 × 20 mm rail, the bag of M4 × 16 machine screws and the 3D printed parts for this pump channel (Fig. 4).

**Step 3: Remove unused parts from the E Plate and mount**

**Fig. 5:**
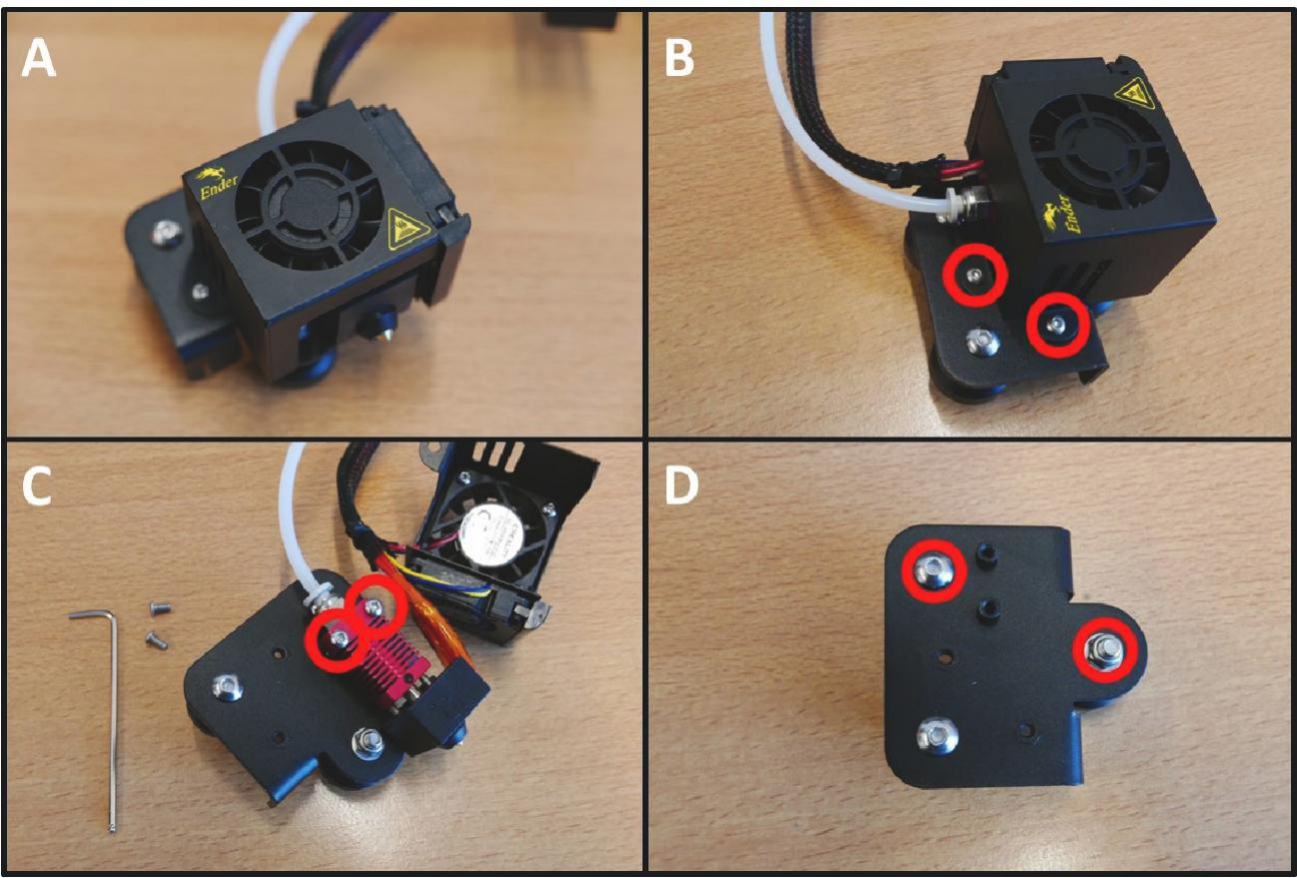
Preparing the E Plate for use in the pump. A) Extruder head overview. B) Removal of hotend shroud screws. These screws need to be saved for later use. C) Removal of the hotend. D) Loosening two screws in preparation for the Slider Mount.

To prepare the E Plate, remove all the unnecessary parts. Start by removing the two M3 screws (save these screws for later use) (Fig. 5B). Now remove the hotend shroud/fan mount. Next, remove the hotend (Fig. 5C). Finally, remove the two screw indicated in red circles (Fig. 5D). The E Plate is now ready to be used in the pump channel.

**Step 4: Install the E Slide Mount on the E Plate**

**Fig. 6:**
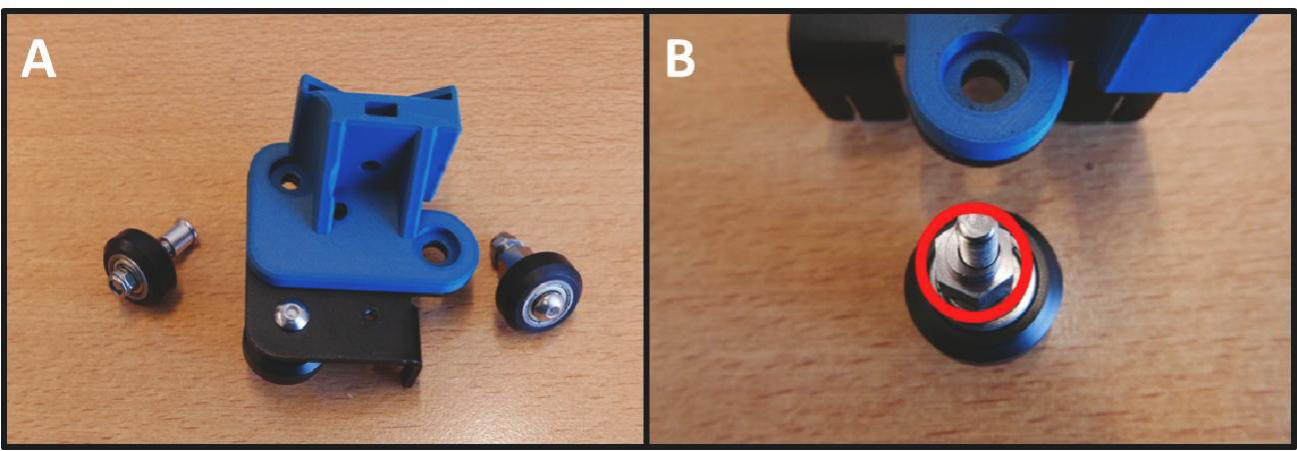
Mounting the E Slide Mount. A) E Slide mount fitted to the E Plate. B) Eccentric adjuster for the guidance mechanism.

Take the E Slide Mount and fit it on the Extruder Plate (Fig. 6A), by replacing the two removed screws from Fig. 5D. Note the eccentric hexagonal adjuster that will be used later to remove slop in rail guidance mechanism.

**Step 5: Install the E Stepper Mount**

**Fig. 7:**
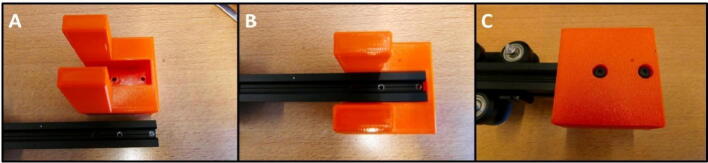
E Stepper Mount installation procedure. A) E Stepper Mount and 20 × 20 rail. B) Rail slides into E Stepper Mount. C) E Stepper Mount secured with M4 × 16 screws.

Install the E Stepper Mount using the M4 × 16 screws (Fig. 4). Slide the mount onto the rail and tighten the screws on the bottom (Fig. 7C).

**Step 6: Install E Slide Mount on rail**

**Fig. 8:**
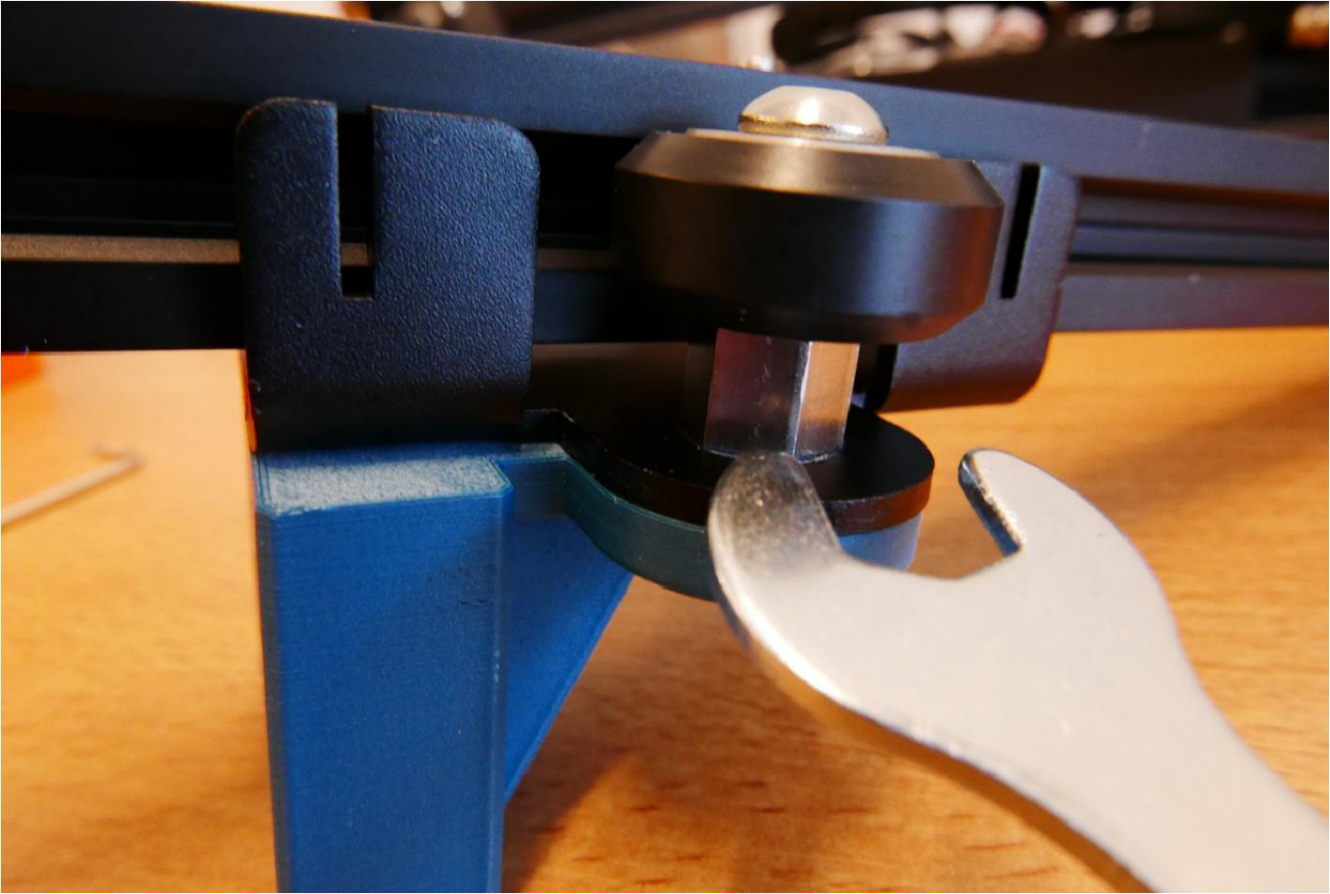
Tension adjustment of the E Slide Mount rollers.

Install the E Slide Mount assembly onto the rail, with the 3D printed mount facing away from the E Stepper Mount. Adjust the tension on the rollers by turning the eccentric hexagonal nut (Fig. 8).

**Step 7: Install the E End Mount**

**Fig. 9:**
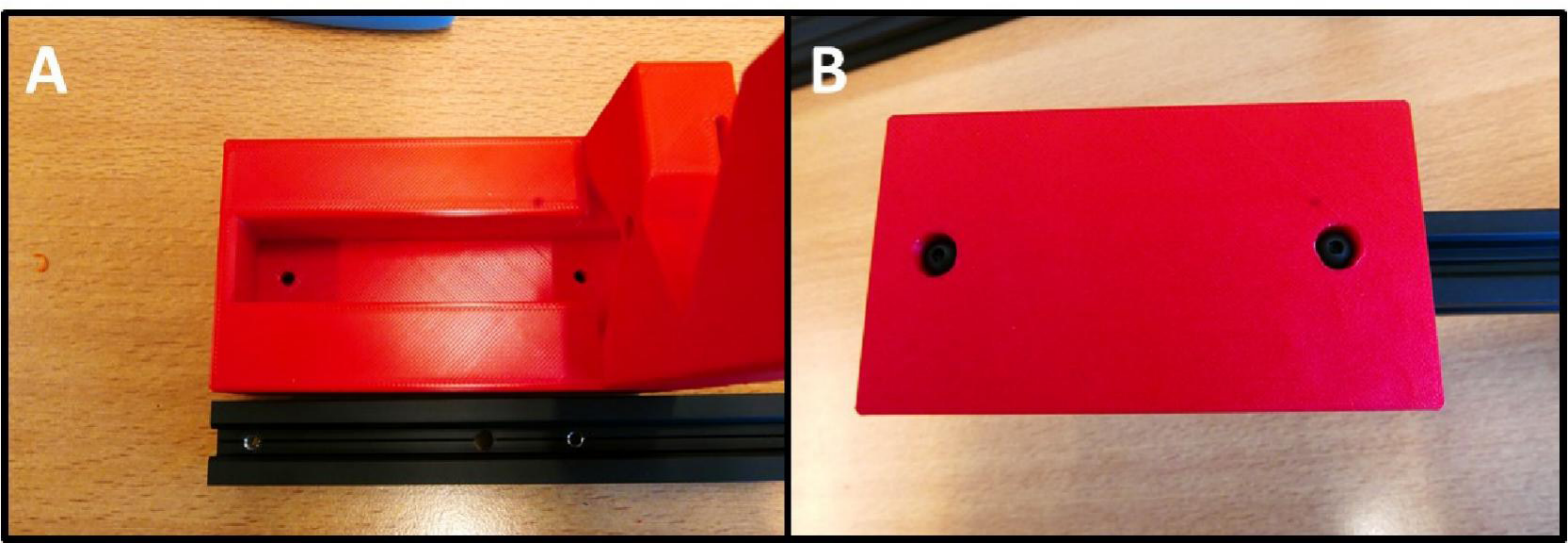
A) E End Mount next to 20 × 20 rail. B) E End Mount fastened using 2 M4 × 16 screws.

Finish the frame of the E Plate Pump by installing the E End Mount. Slide the mount on the rail (Fig. 9A) and use the remaining M4 × 16 screws to attach the mount (Fig. 9B).

**Step 8: Remove unnecessary parts from stepper motor**

**Fig. 10:**
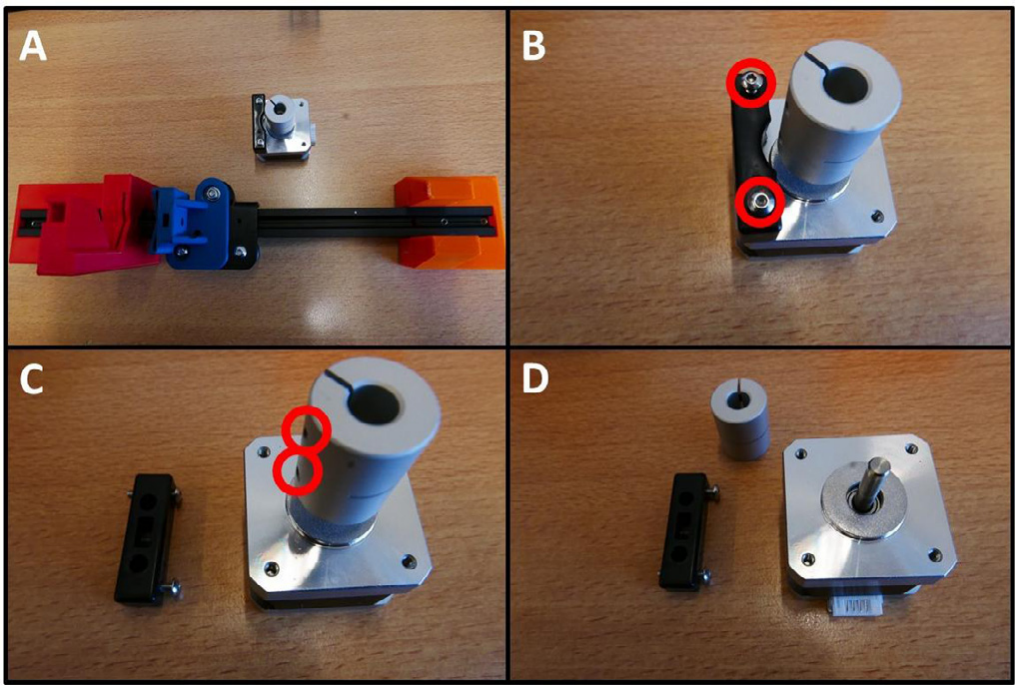
Preparing the stepper motor for installation. A) Overview of the E Plate pump and stepper. B) Removal of bracket. C) Removal of shaft coupler. D) Stepper ready for use in pump.

Take the stepper motor shown in Fig. 10A. Remove the screws circled in red (Fig. 10B). Then, loosen the grub screws in red (Fig. 10C) and remove the coupler. The stepper motor is now ready (Fig. 10D).

**Step 9: Install stepper motor on the E Stepper Mount**

**Fig. 11:**
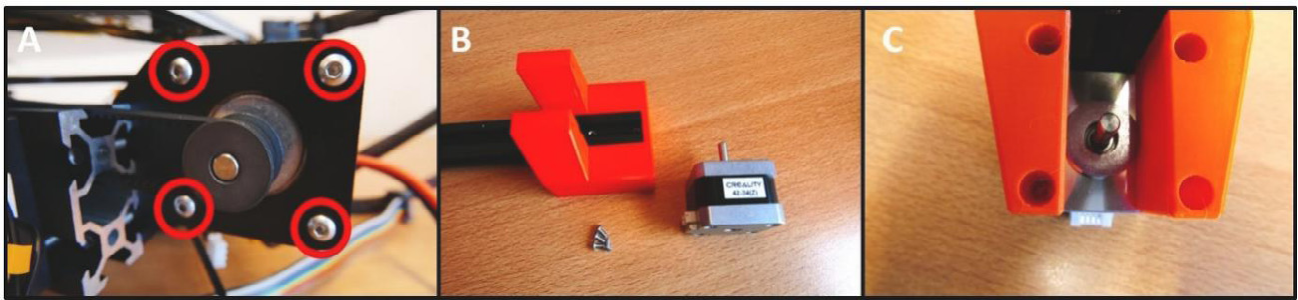
Stepper motor mounting. A) Gathering M3 × 6 screws from the Y stepper. B) All parts to be combined. C) Stepper motor mounted on the E Stepper Mount.

The stepper motor is now to be installed on the E Stepper Mount. For the installation, 4 M3 × 6 screws are required. These can be taken from the Y stepper motor, shown in Fig. 11A. Save the Y stepper for later use. Fig. 11B shows the E Stepper Mount, the stepper motor to be installed and the M3 × 6 screws. These screws can be inserted in the counterbored holes of the E Stepper Mount and tightened, to affix the stepper motor.

**Step 10: Cut the M5 threaded rod to size**

**Fig. 12:**
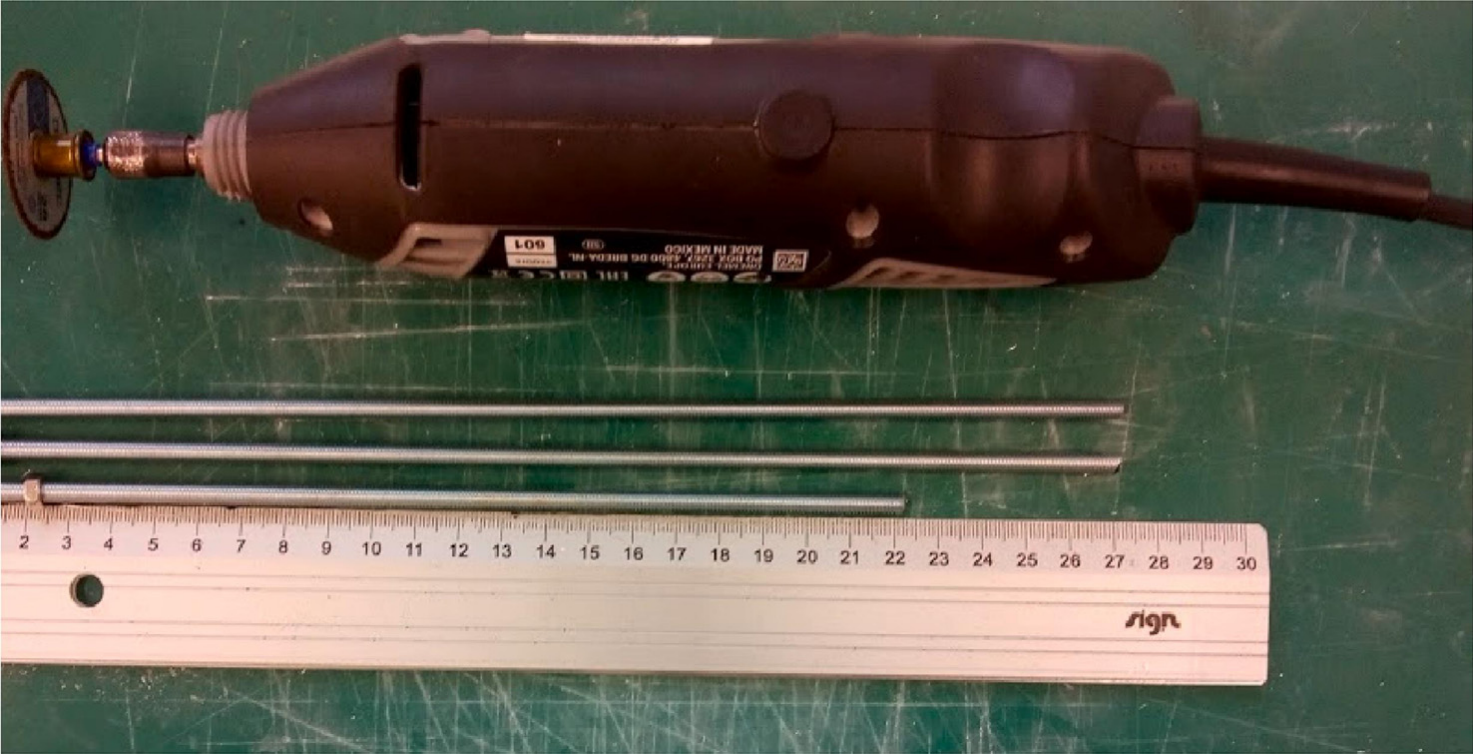
M5 Threaded rods cut to length. Two 27 cm rods for the X Plate and X Motor Plate pumps, 22 cm rod for the E Plate pump.

Make sure to wear appropriate personal protective equipment when using cutting tools (eye protection, hearing protection, dust extraction). For the drive rod, an M5 threaded rod needs to be cut to size. This can be done using a hacksaw or a power tool such as a cutoff wheel. Before starting the cut, thread on an M5 nut onto the rod. Cut the rod and clean off the sharp end with a file or sandpaper. After cutting and filing, the nut can be threaded over the cut end. This will straighten out the threads. 2 rods of 27 cm and 1 rod of 22 cm are needed to complete all three pumps (Fig. 12).

**Step 11: Install the threaded rod**

**Fig. 13:**
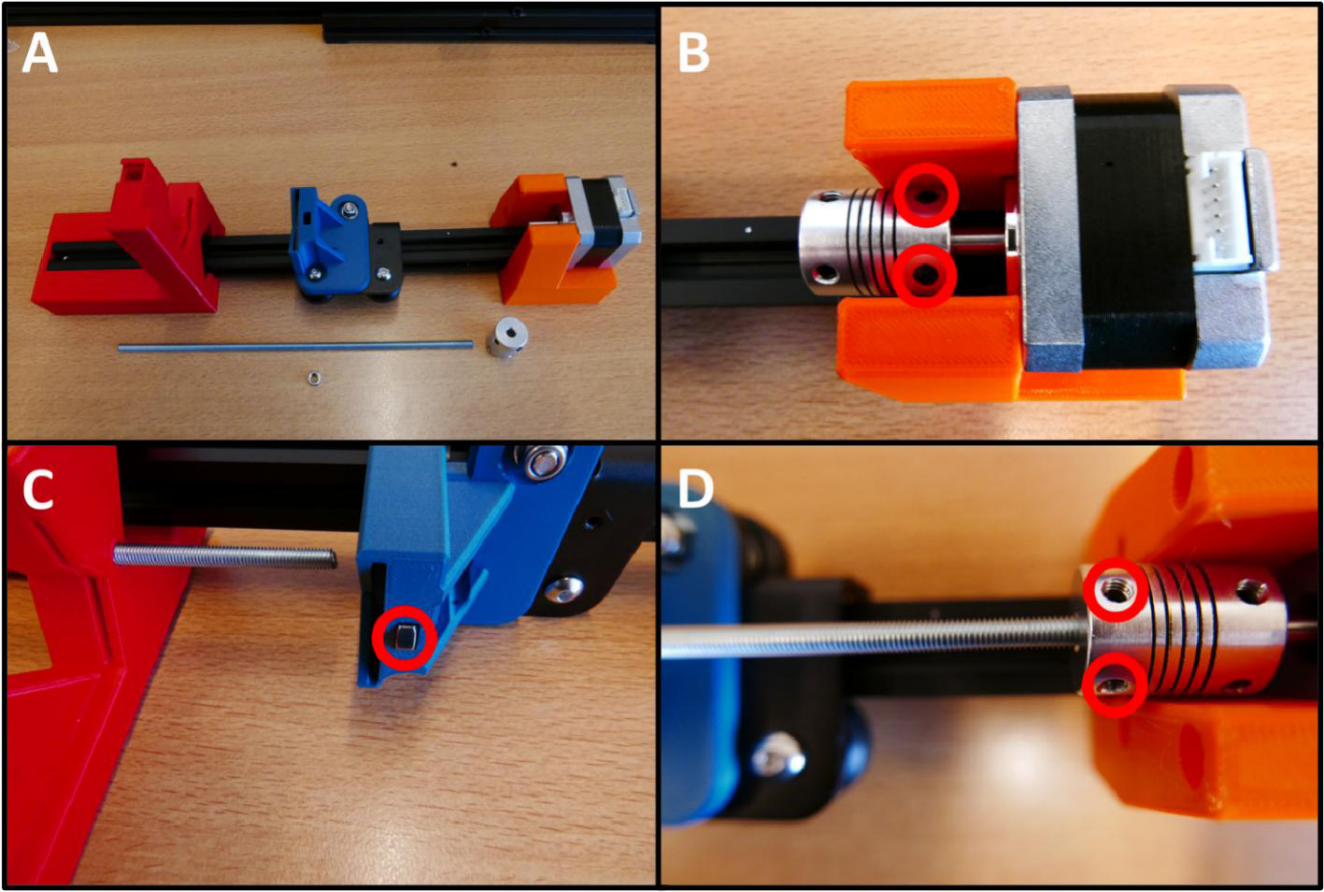
Installation of the threaded rod and shaft coupler. A) All components laid out. B) Shaft coupler installation. C) Fixing the threaded rod to the E Slide Mount using an M5 nut. D) Threaded rod clamped in the shaft coupler.

To install the threaded rod, take the 22 cm threaded rod, 1 M5 nut and a shaft coupler (Fig. 13A). Install the shaft coupler onto the stepper motor by tightening the two grub screws (Fig. 13B). Slide the threaded rod through the E End Mount, up to the E Slide Mount. Lay the assembly on its side and push in the nut, centering it with the through-hole for the rod (Fig. 13C). Thread the rod through the nut, all the way up to the shaft coupler. Tighten the grub screws to install the threaded rod (Fig. 13D).

**Step 12: Collect the springs for the syringe clamp**

**Fig. 14:**
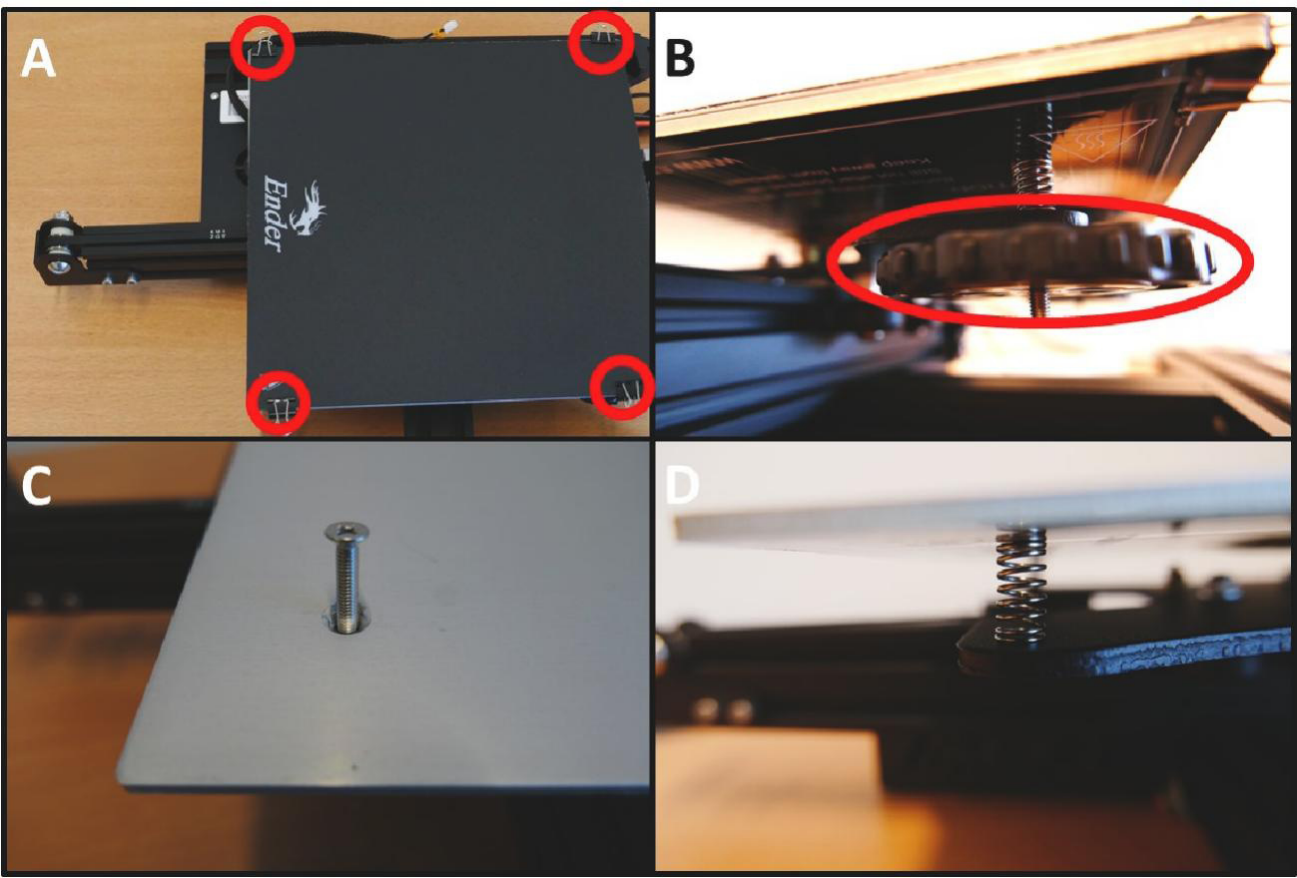
Obtaining the springs for the syringe clamp mechanism. A) Remove the spring clips holding the printbed cover. B) Remove the plastic handwheel. C) Remove the machine screw. D) The spring can now be removed and used in the pump.

To clamp the syringes in place, the springs from the bed levelling mechanism will be used. Start by removing the clips and printbed cover (Fig. 14A). Unscrew the large plastic wheel (Fig. 14B). Next, remove the machine screw to free up the spring (Fig. 14C). The spring can now be removed (Fig. 14D). Repeat this process to collect 3 springs in total.

**Step 13: Install the syring clamp mechanism**

**Fig. 15:**
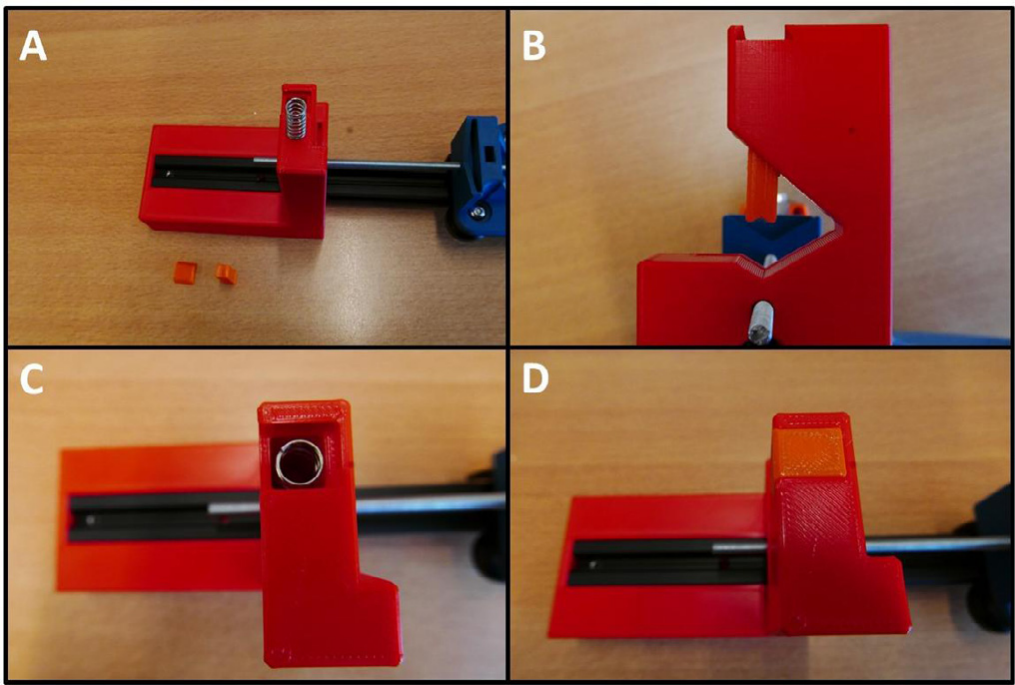
Fitting the syringe clamping mechanism. A) The necessary parts: Spring, Clamp Bar and Dovetail Cap. B) Clamp Bar installed in the slot. C) Spring placed on top of the Clamp Bar. D) The slot closed off with a Dovetail Cap, securing the mechanism in place.

The syringe clamping mechanism can now be installed. This mechanism consists of a Clamp Bar, Dovetail Cap and one of the springs (Fig. 15A). Slide the clamp bar into the slot on top of the E End Mount (Fig. 15B). Next, place the spring on top, in the square hole (Fig. 15C). Finally, close the top with a Dovetail Cap. The pump channel is now ready to be used. Different length Clamp Bars can easily be exchanged to accommodate different syringe diameters.

**Step 14: Collecting the 40 × 40 rails**

**Fig. 16:**
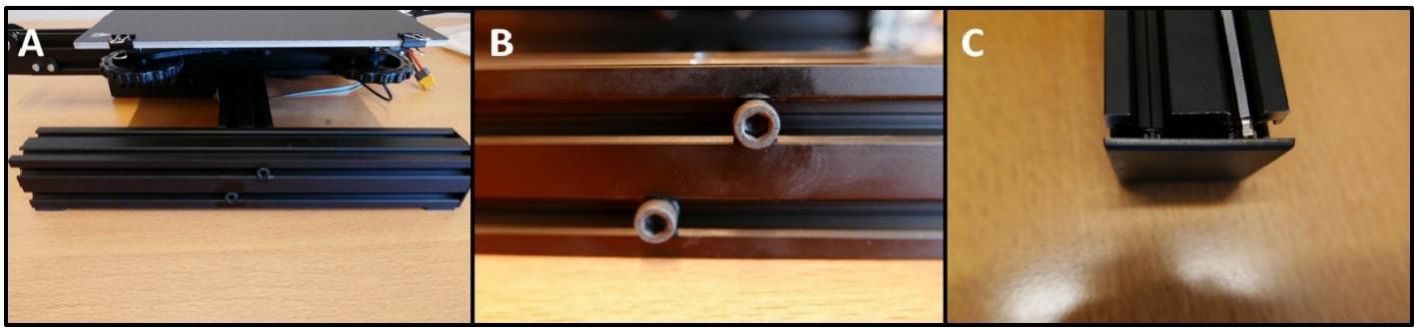
Gathering the 40 × 40 rail. A) The rail still mounted to the Ender 3 base. B) Loosen the M5 × 45 screws to remove the rail. C) Using a hex wrench, the end cap can be pushed out.

To build the X Motor Plate pump, a 40 × 40 rail is needed from the Ender 3 frame (Fig. 16A). Loosen the two M5 × 45 screws that are holding this piece and save the screws (Fig. 16B). Use one of the hex wrenches to push out the end cap (Fig. 16C).

**Fig. 17:**
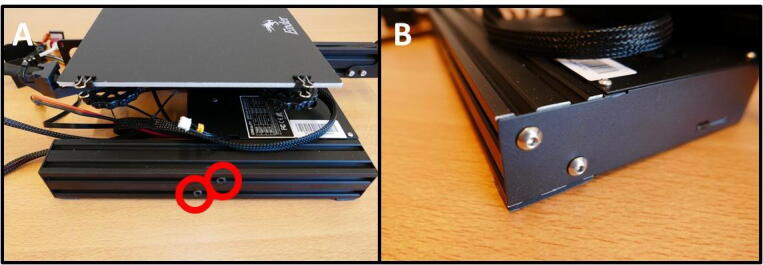
Gathering the second 40 × 40 rail. A) Removing the M5 × 45 screws. B) Removing the electronics case.

For the X Plate pump another 40 × 40 rail is need, from the other side of the base. Loosen the M5 × 45 screws (Fig. 17A) and save them. Then, also remove the electronics case by removing the two screws (Fig. 17B). Save these screws as well. Remove the end cap on the rail like in Fig. 16C.

**Step 15: Collect the parts for the X Motor Plate Pump**

**Fig. 18:**
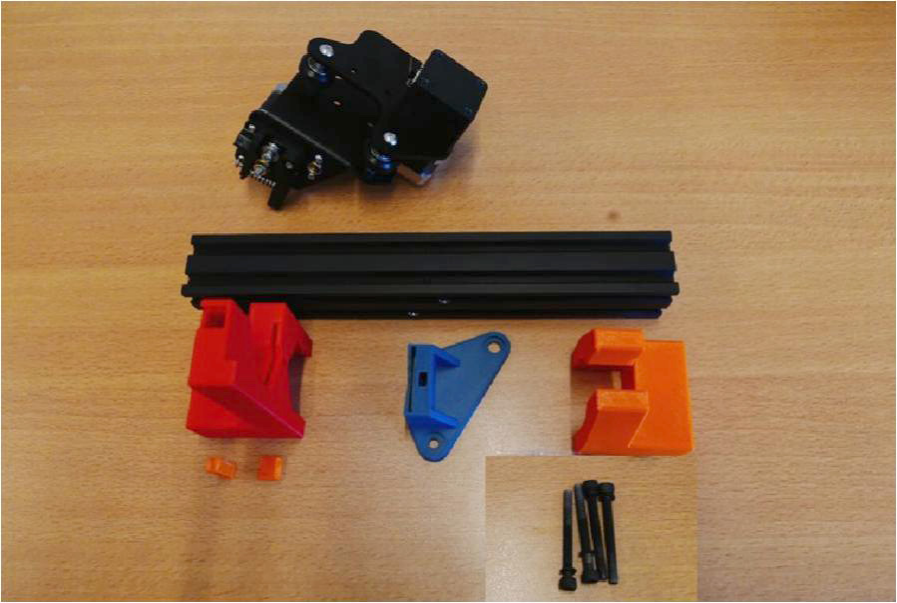
The parts needed to build the X Motor Plate pump.

To build the X Motor Plate Pump, take the X Motor Assembly, 40 × 40 rail, X X Motor End Mount, X Motor Plate Slide Mount, X X Motor Stepper Mount, Clamp Bar, Dovetail cap and four M5 × 45 screws (Fig. 18).

**Step 16: Remove unnecessary parts from X Motor assembly**

**Fig. 19:**
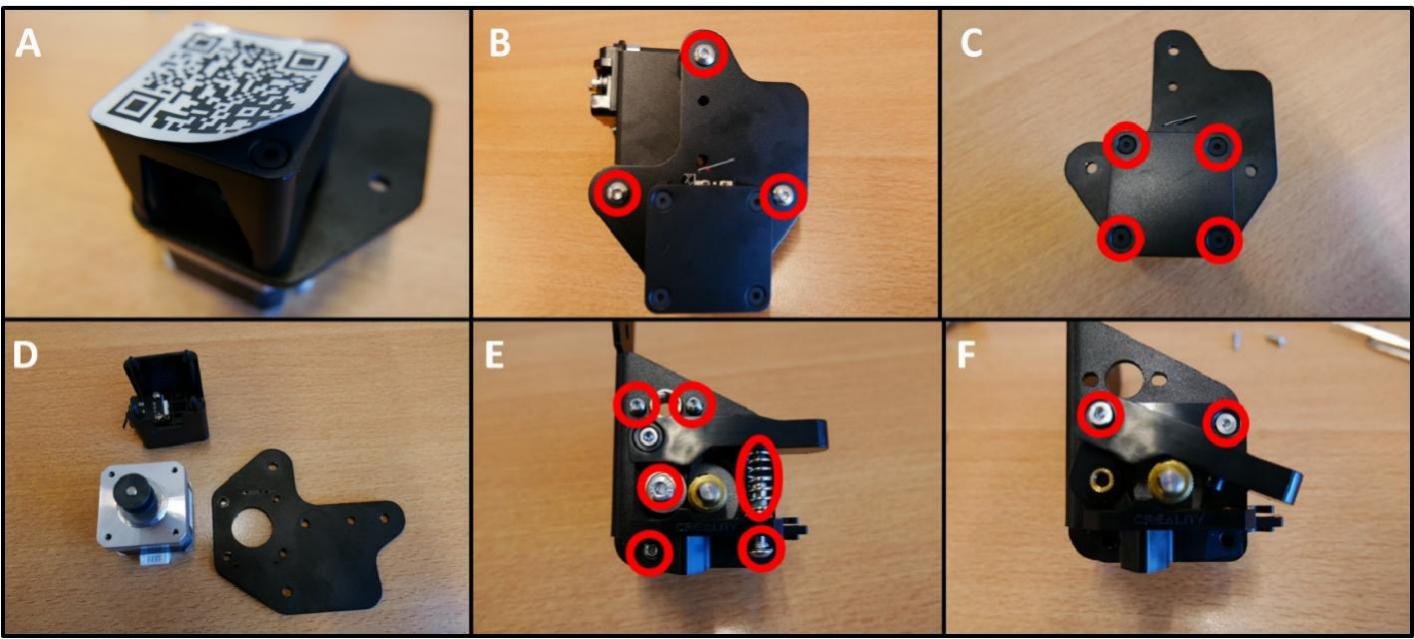
Procedure for disassembling the X Motor Assembly. A) Peel away the sticker. B) Remove the three roller screws. C) Remove the pulley cover. D) The freed up parts. Save the stepper motor. E) Remove all screws and the spring indicated in red. F) Remove the screws holding the filament clamp in place. (For interpretation of the references to colour in this figure legend, the reader is referred to the web version of this article.)

**Fig. 20:**
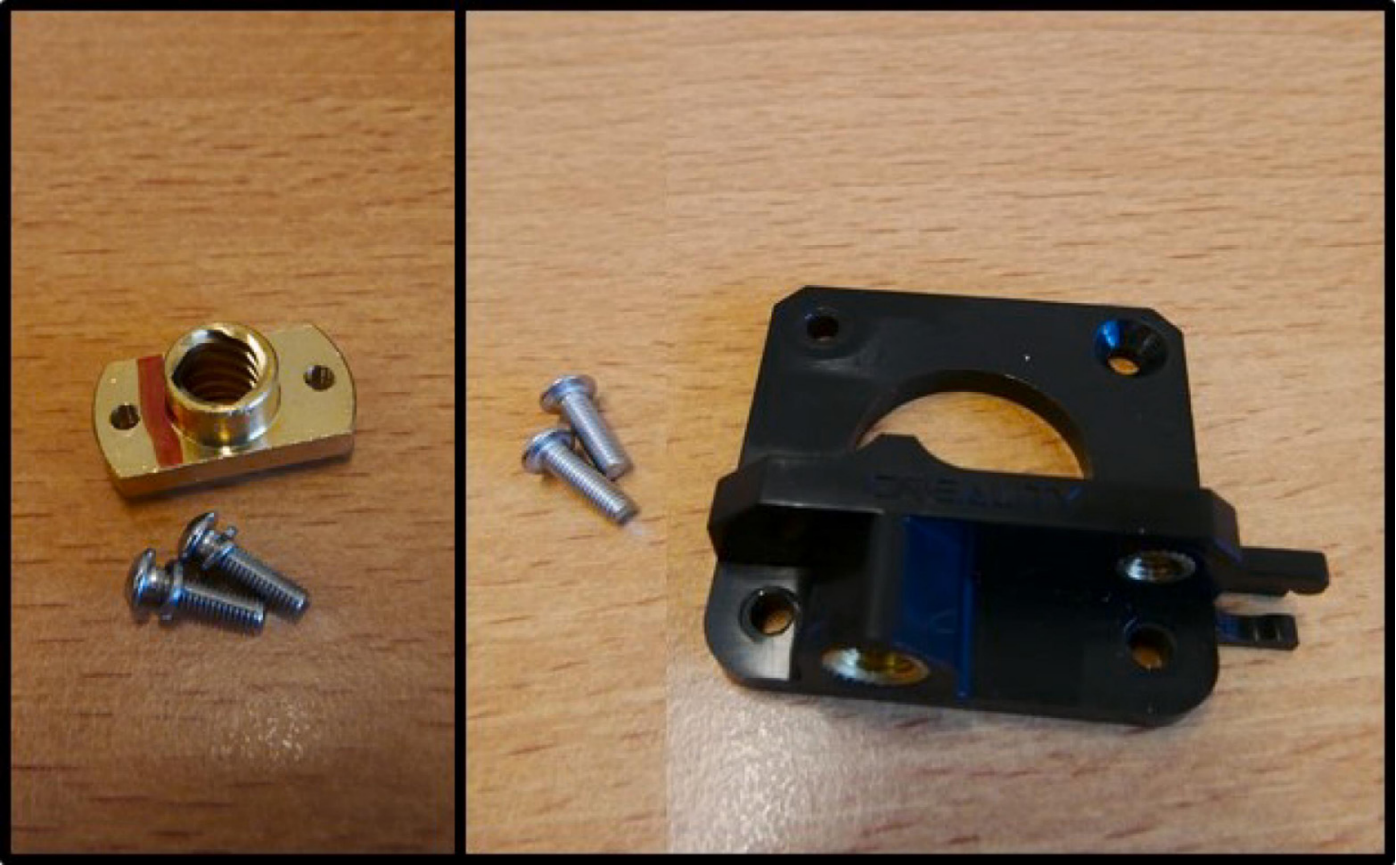
M3 × 10 screws to be used for later steps in the build guide.

The X Motor assembly consists of many parts, that need to be removed in order to use the X Motor Plate in the pump. Start by removing the sticker covering the screws (Fig. 19A). Next, remove the roller screws holding the two plates together (Fig. 19B). Remove the screws from the pulley cover (Fig. 19C). Save the stepper motor for later use (Fig. 19D). Next, remove all the screws indicated in red in Fig. 19E, remove the spring as well. Finally, remove the last two screws holding the filament clamping mechanism in place (Fig. 19F). Save four M3 × 10 screws for later use (Fig. 20).

**Step 17: Install the X Motor Slide Mount on the X Motor Plate**

**Fig. 21:**
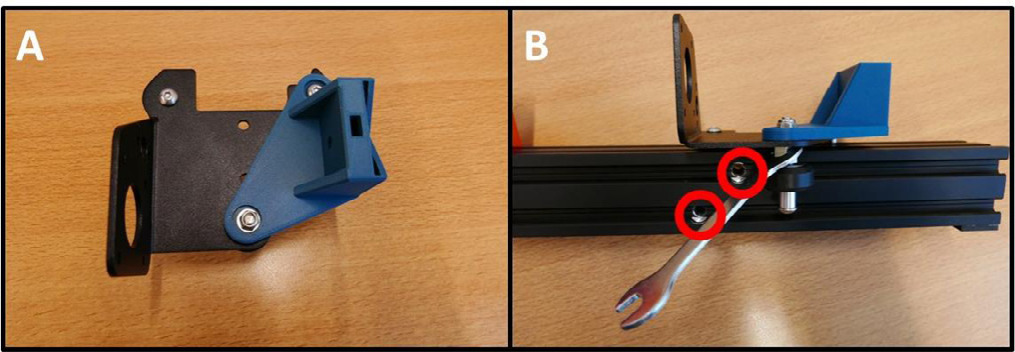
Installing the X Motor Slide Mount. A) 3D printed part mounted on the X Motor Plate. B) Fitting the assembly on the rail.

Take the angle bracket X Motor Plate and mount the X Motor Slide Mount, similar to step 4 (Fig. 21A). Slide the mount onto the 40 × 40 rail and adjust the tension. Make sure the countersunk holes on the rail are on the side of the single guide wheel (Fig. 21B).

**Step 18: Removing the press-fit pulleys from the X and Y Motor**

**Fig. 22:**
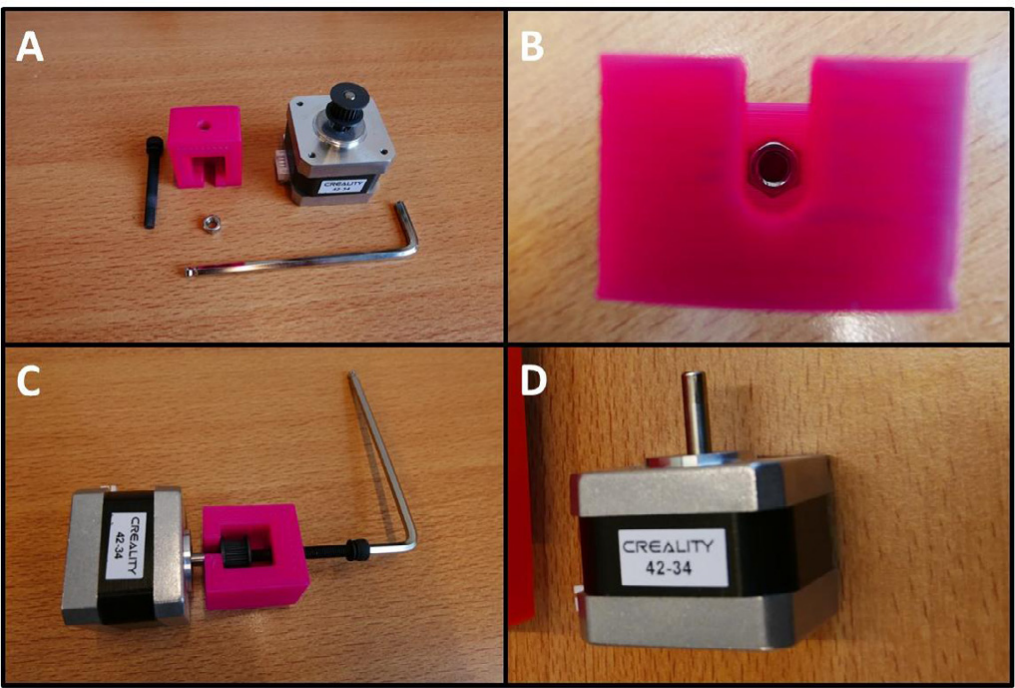
Stepper pulley removal process. A) Overview of the tool, parts and stepper. B) Fitting the M5 nut in the tool. C) Removing the pulley with the tool. D) Stepper motor ready for use in the pump.

Take the X Stepper from step 16 and Y Stepper from step 9. They have press-fit pulleys that need to be removed. This can be accomplished by using a pulley removal tool. A 3D printed version is available from https://www.thingiverse.com/thing:3593964. Print this tool at 100% infill (fully solid). To use the tool, an M5 nut and M5 × 45 screw can be used (Fig. 22A). Press the M5 nut into the hexagonal slot (Fig. 22B) and thread in the screw. Slide the tool around the pulley and align the screw with the stepper shaft. Turn the screw to apply pressure on the shaft (Fig. 22C). With the pulley removed, the stepper is ready to be used in the pump (Fig. 22D). Repeat this process for the second stepper.

**Step 19: Install the X X Motor Stepper Mount**

**Fig. 23:**
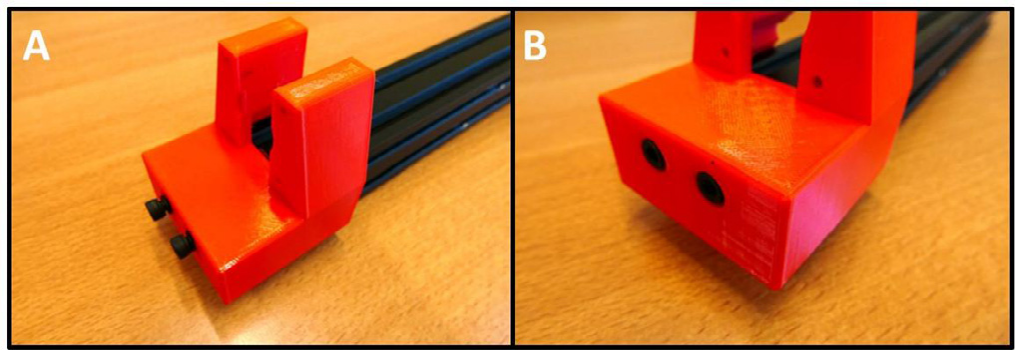
Installing the X X Motor End Mount. A) Fitting the mount on the rail. B) Fully installed.

Slide the X X Motor Stepper Mount on the 40 × 40 rail and insert the M5 × 45 screws (Fig. 23A). Fasten the screw, the X X Motor Stepper Mount is now attached (Fig. 23B).

**Step 20: Install the X X Motor End Mount**

**Fig. 24:**
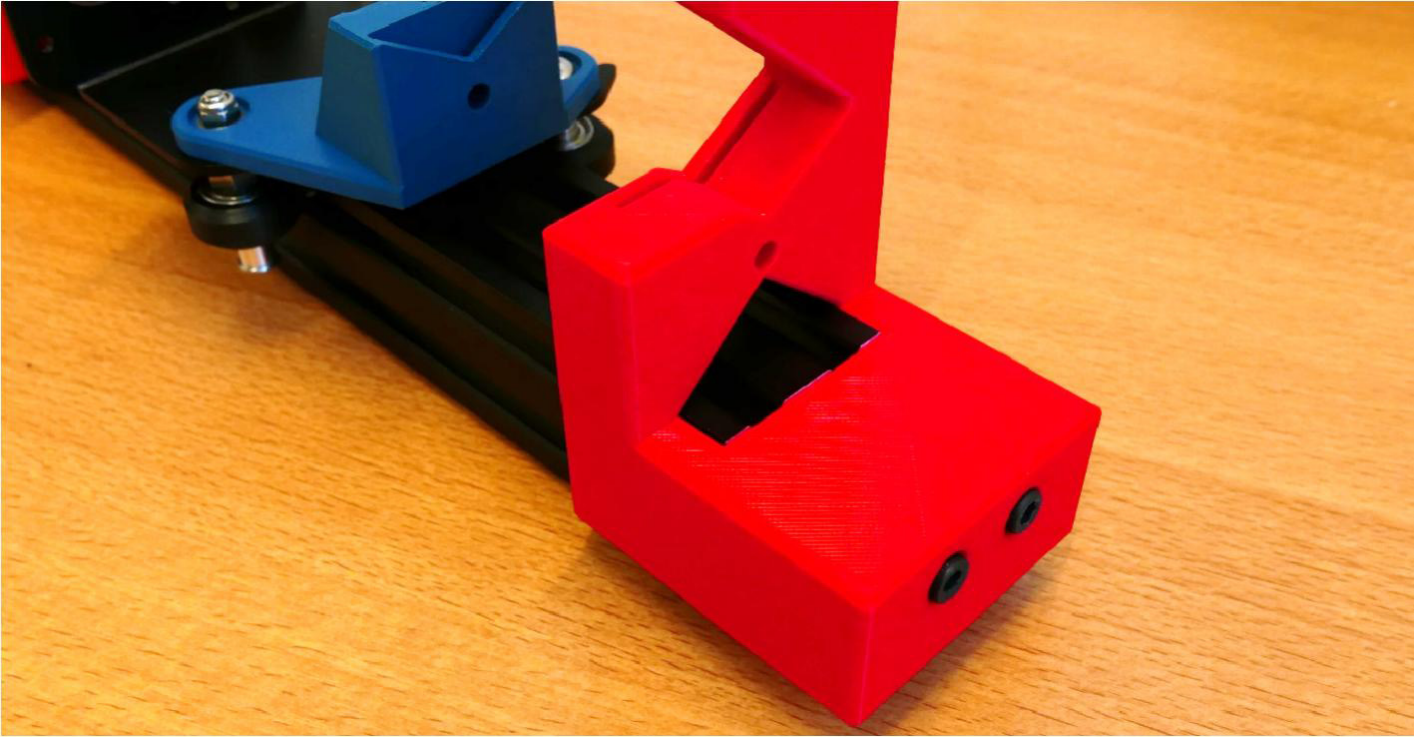
The installed X X Motor End Mount.

Place the X X Motor End Mount on the opposite end of the 40 × 40 rail. Fasten the part with two M5 × 45 screws (Fig. 24).

**Step 21: Install the stepper motor**

**Fig. 25:**
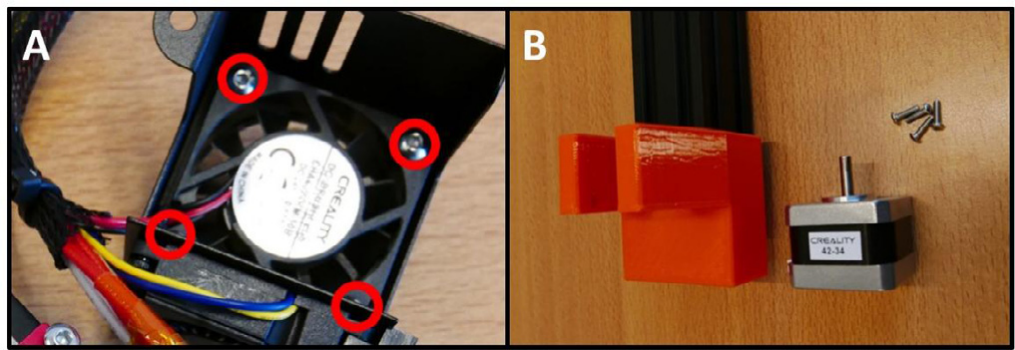
The stepper motor will be mounted with M3 × 10 screws. A) Take the M3 × 10 screws from the fan mounted on the extruder head. B) Ready to install the stepper motor.

Install the stepper motor on the X X Motor Stepper Mount. Take 4 M3 × 1-screws by dismounting the Extruder Fan from the shroud (Fig. 25A). Use these screws to install the stepper motor (Fig. 25B).

**Step 22: Install the threaded rod**

**Fig. 26:**
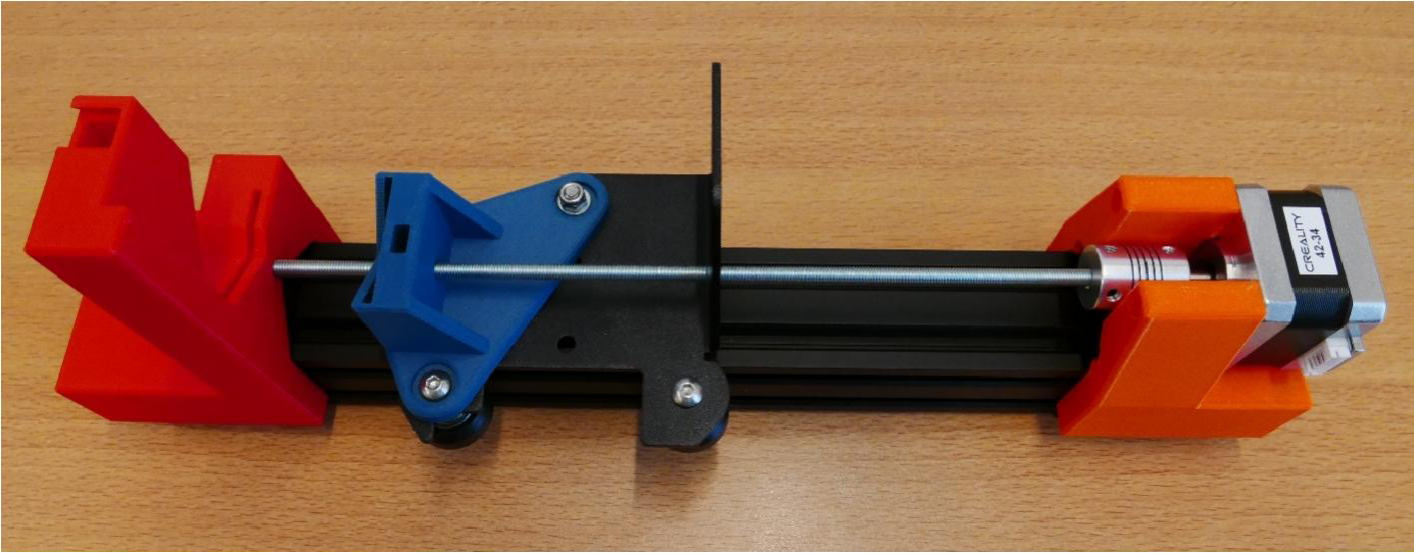
Installed threaded rod in the X Motor Plate pump.

Take one of the 27 cm threaded rods from step 10 and install it in the X Motor Plate pump, follow the procedure from step 11. The end result looks like Fig. 26.

**Step 23: Install the syringe clamp mechanism**

**Fig. 27:**
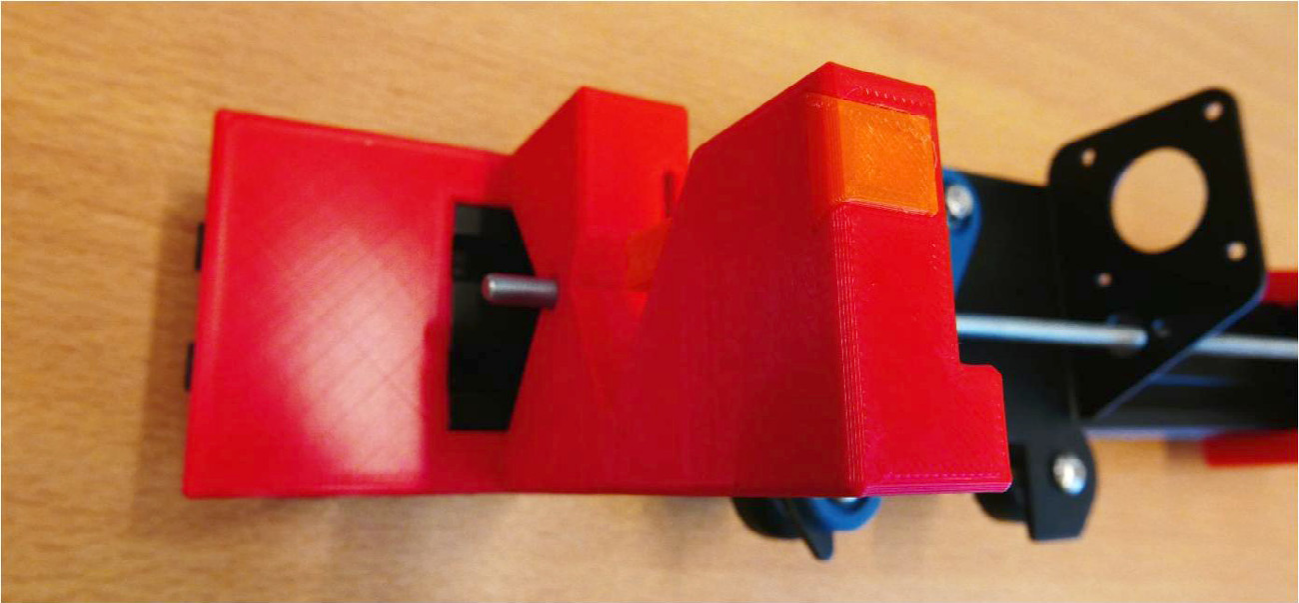
Installed syringe clamp mechanism.

Follow step 13 and install the syringe clamp mechanism. The X Motor Plate pump channel is now finished (Fig. 27).

**Step 24: Collect the parts for the X Plate Pump**

**Fig. 28:**
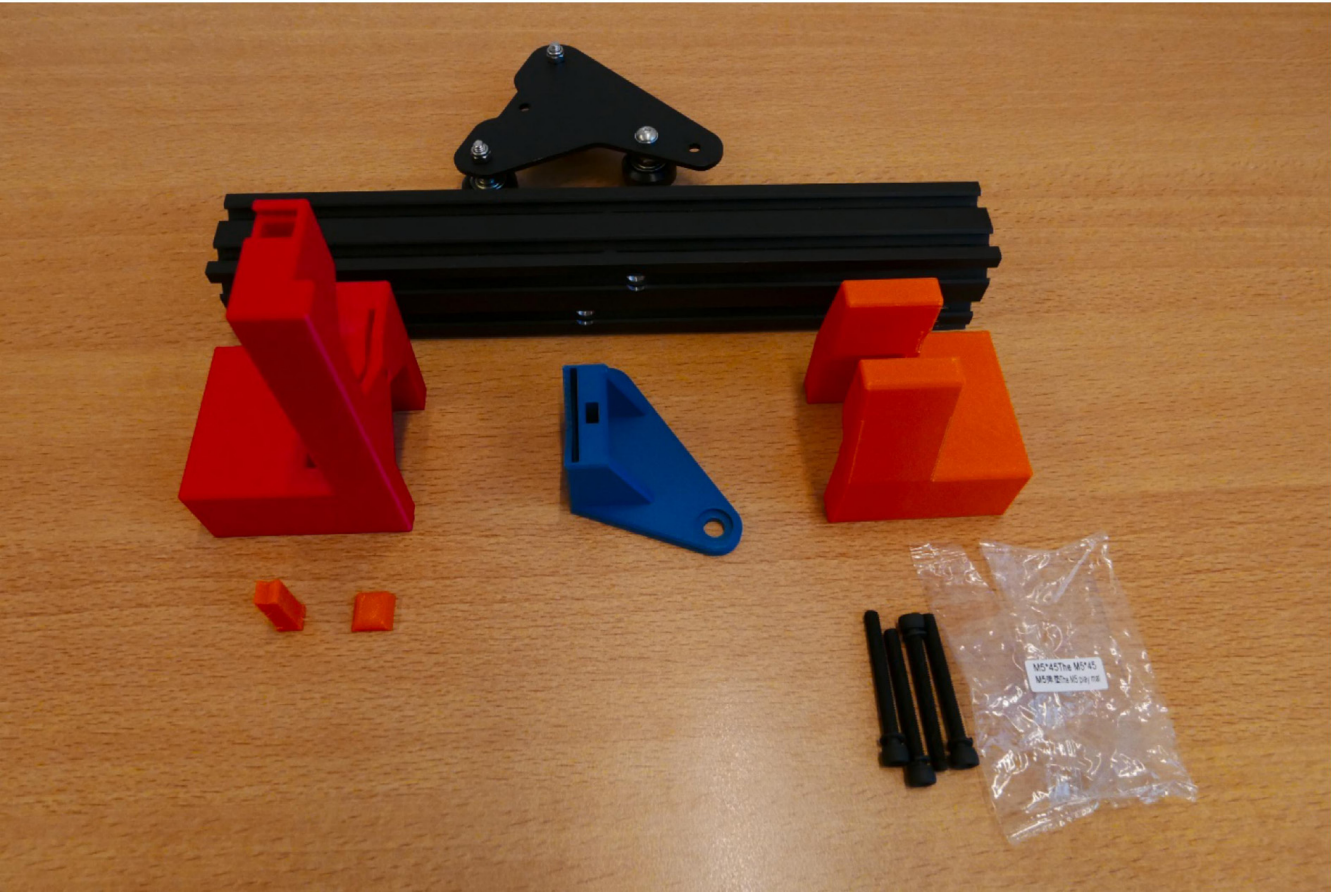
All the parts needed to build the X Plate Pump frame.

To build the X Plate Pump, Take the X Plate, 40 × 40 rail, X X Motor End Mount, X Plate Slide Mount, X X Motor Stepper Mount, Clamp Bar, Dovetail Cap and bag with four M5 × 45 screws (Fig. 28).

**Step 25: Build the X Plate Pump according to previous steps**

**Fig. 29:**
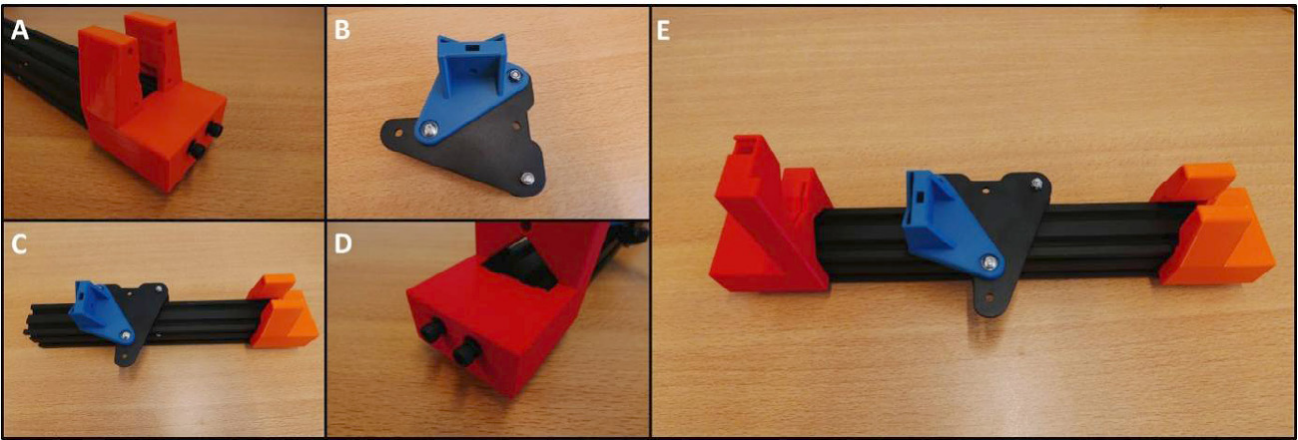
Building the X Plate Pump frame. A) X X Motor Stepper Mount installation. B) X Slide Mount orientation. C) Rail assembly D) Fastening the M5 × 45 screws on the X X Motor End Mount. E) Completed X Plate Pump frame.

The X Plate pump construction is very similar to the X Motor Plate Pump. Mount the X X Motor Stepper Mount like in step 19 (Fig. 29A). Mount the X Slide Mount according to step 17, in the correct orientation (Fig. 29B) and slide onto rail (Fig. 29C). Install the X X Motor End Mount following step 20 (Fig. 29D). The X Plate Pump frame is now finished (Fig. 29E).

**Step 26: Install the stepper motor**

**Fig. 30:**
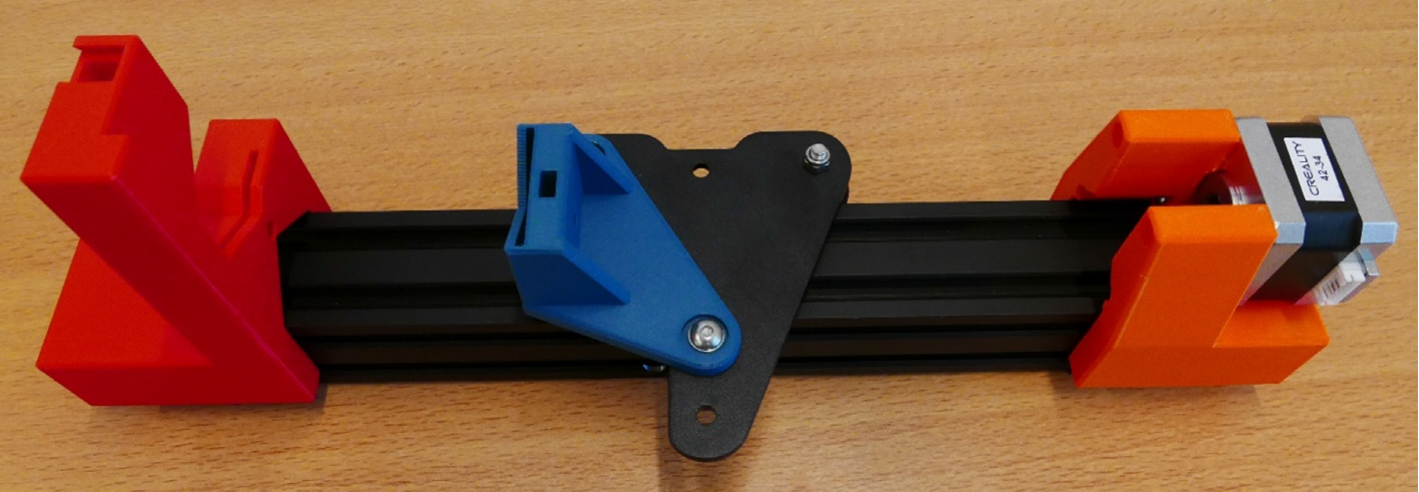
Stepper motor installed in the X Plate Pump frame.

Install the stepper motor using the four M3 × 10 screws that were saved in step 16, according to the procedure of step 21 (Fig. 30).

**Step 27: Install the threaded rod**

**Fig. 31:**
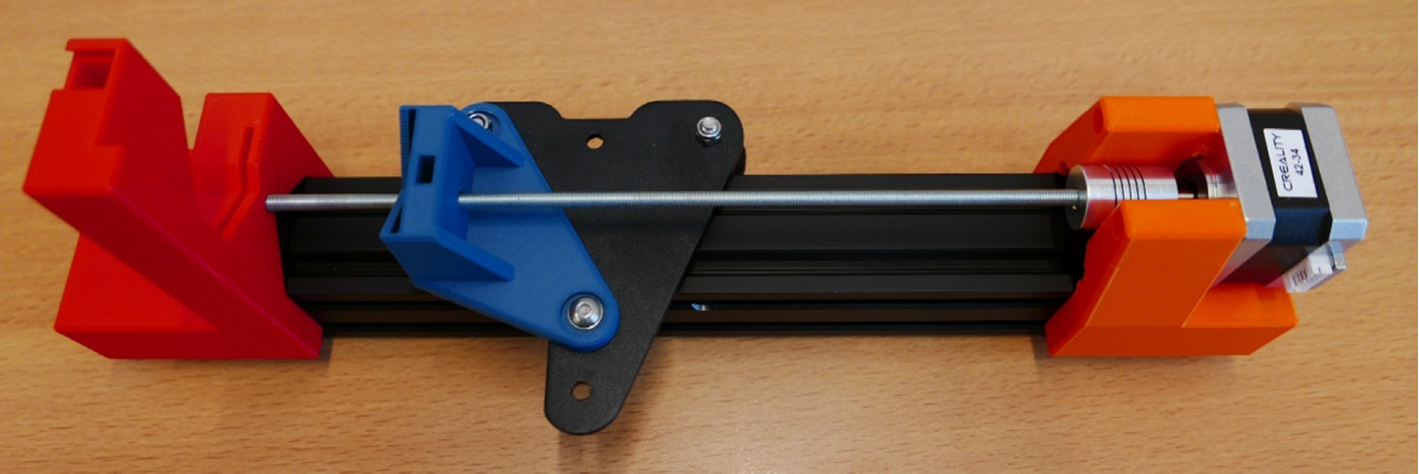
Threaded rod installed in the X Plate Pump.

Take one of the 27 cm threaded rods from step 10 and install it in the X Plate pump, follow the procedure from step 11. The end result looks like Fig. 31.

**Step 28: Install the syringe clamp mechanism**

**Fig. 32:**
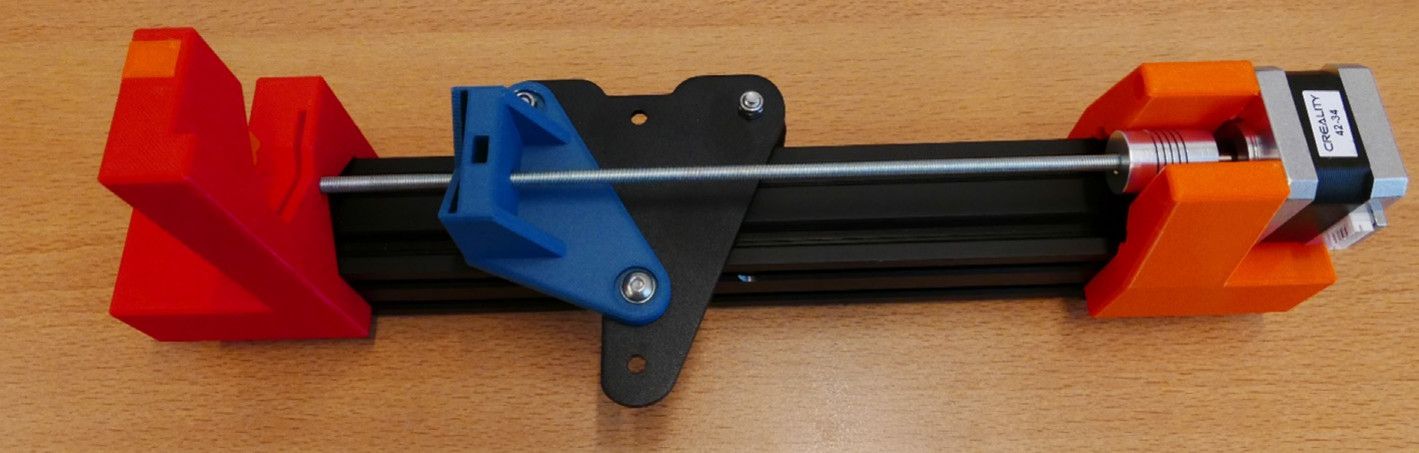
The completed X Plate Pump.

Follow step 13 and install the syringe clamp mechanism. The X Plate pump channel is now finished (Fig. 32).

**Step 29: Detaching the Electronics Box**

**Fig. 33:**
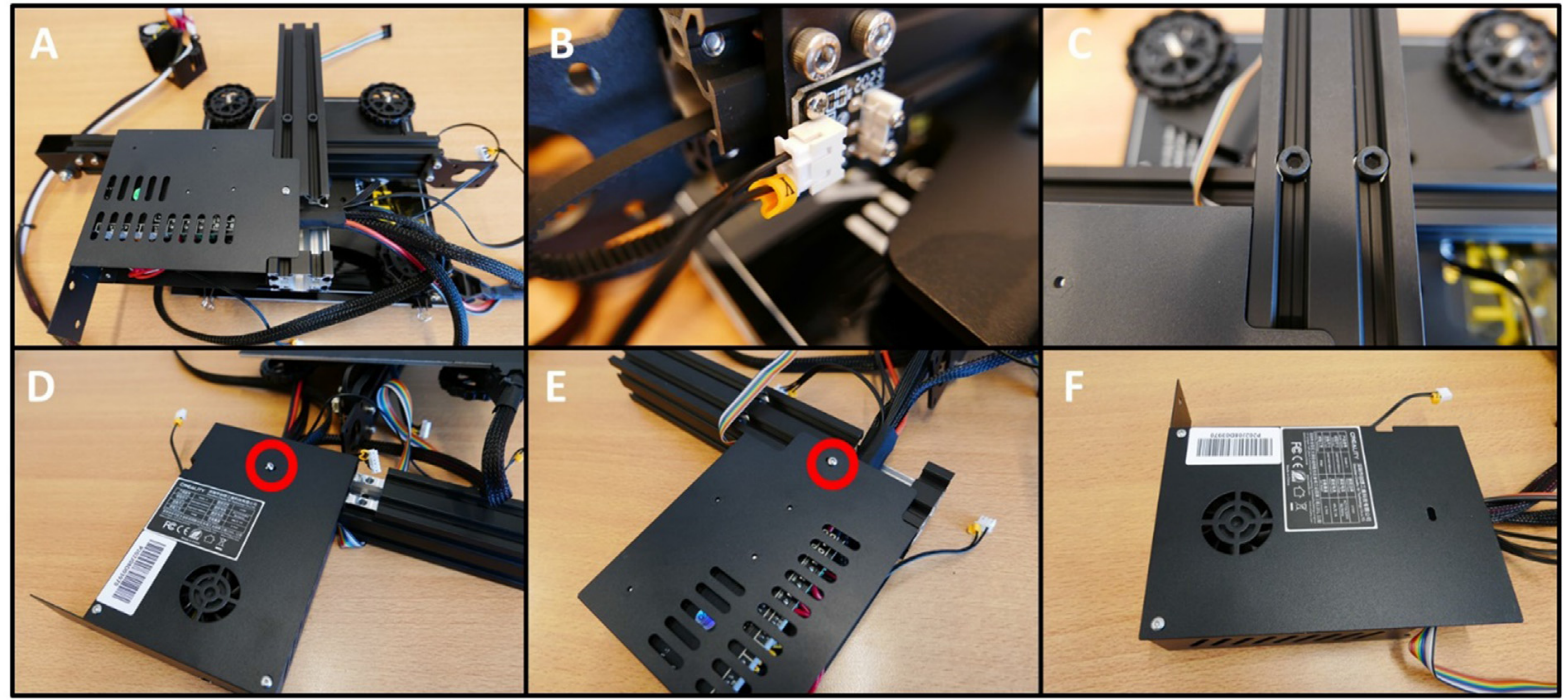
Removing the Electronics Box from the Ender 3 base. A) Overview of the underside of the base. B) Y limit switch connector. C) M5 × 45 screws, holding the base to the buildplate. D) One of the two mounting screws. E) Second mounting screw. F) The detached Electronics box.

Take the remainder of the Ender 3 base and flip it over (Fig. 33A). Detach the cable from the Y limit switch (Fig. 33B). Remove the M5 × 45 screws to free up the 40 × 40 rail (Fig. 33C). Keep the rail for later use. Remove the screw on the fan-side of the Electronics Box (Fig. 33D). Remove the screw on the other side of the Electronics Box (Fig. 33E). The Electronics Box is now ready to open up.

**Step 30: Removing the Electronics Board and cable restrictions**

**Fig. 34:**
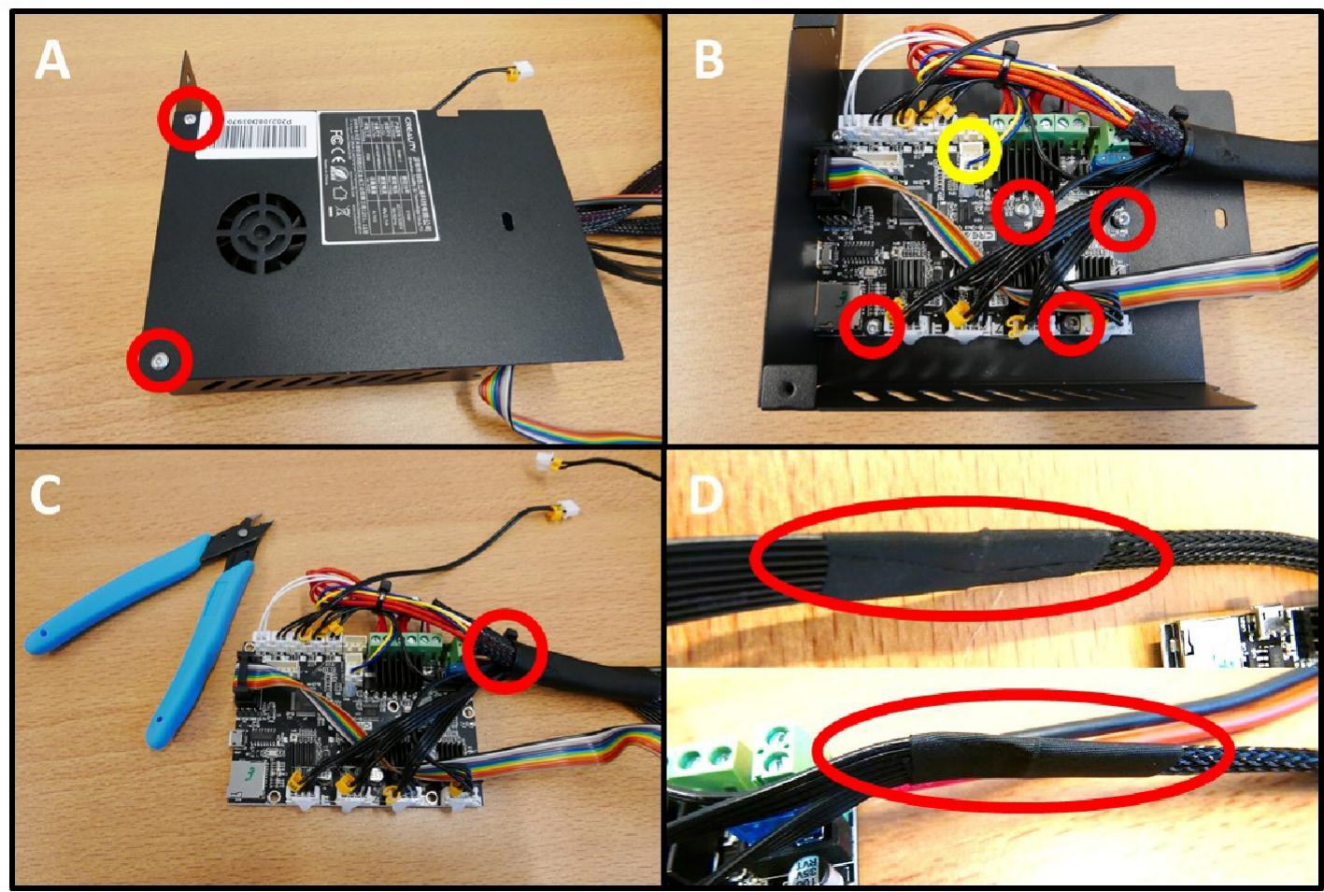
Freeing up the cable assembly to remove redundant cabling. A) Opening the Electronics Box. B) Removing the board mounting screws. Yellow circle indicates the removed fan connector. C) Cutting the zip tie. D) Removing the tape at the braid ends. (For interpretation of the references to colour in this figure legend, the reader is referred to the web version of this article.)

All the redundant cables from the Electronics Board have to be removed. These are cables for the limit switches, bed and hotend heater, hotend fan and extruder motor, which are all not used in this project. To do this, open up the Electronics Box (Fig. 34A). Remove the screws holding the board in place (Fig. 34B). Note the yellow circle, indicating the fan connector. The fan is temporarily removed to facilitate this and the next step. Next, cut the zip tie on the cable assembly (Fig. 34C). Finally, remove the tape at both ends of the braided cable shield (Fig. 34D).

**Step 31: Removing redundant cables**

**Fig. 35:**
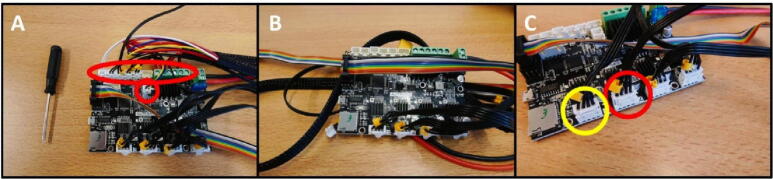
Removal of redundant cables. A) All cables to remove are indicated in red. B) After cable removal. C) Remove the Z stepper cable (red) and replace with the E stepper cable (yellow). (For interpretation of the references to colour in this figure legend, the reader is referred to the web version of this article.)

As mentioned in the previous step, there are several unused, redundant cables that are not used in this project. These include the cables along the top row of the Electronics Board which can be removed, as well as the blue/yellow cable. These are indicated in red (Fig. 35A). The board after removal of these cables is shown in Fig. 35B. Next, remove the short Z stepper cable and replace it with the long E stepper cable.

**Step 32: Collect the components for the Control Unit**

**Fig. 36:**
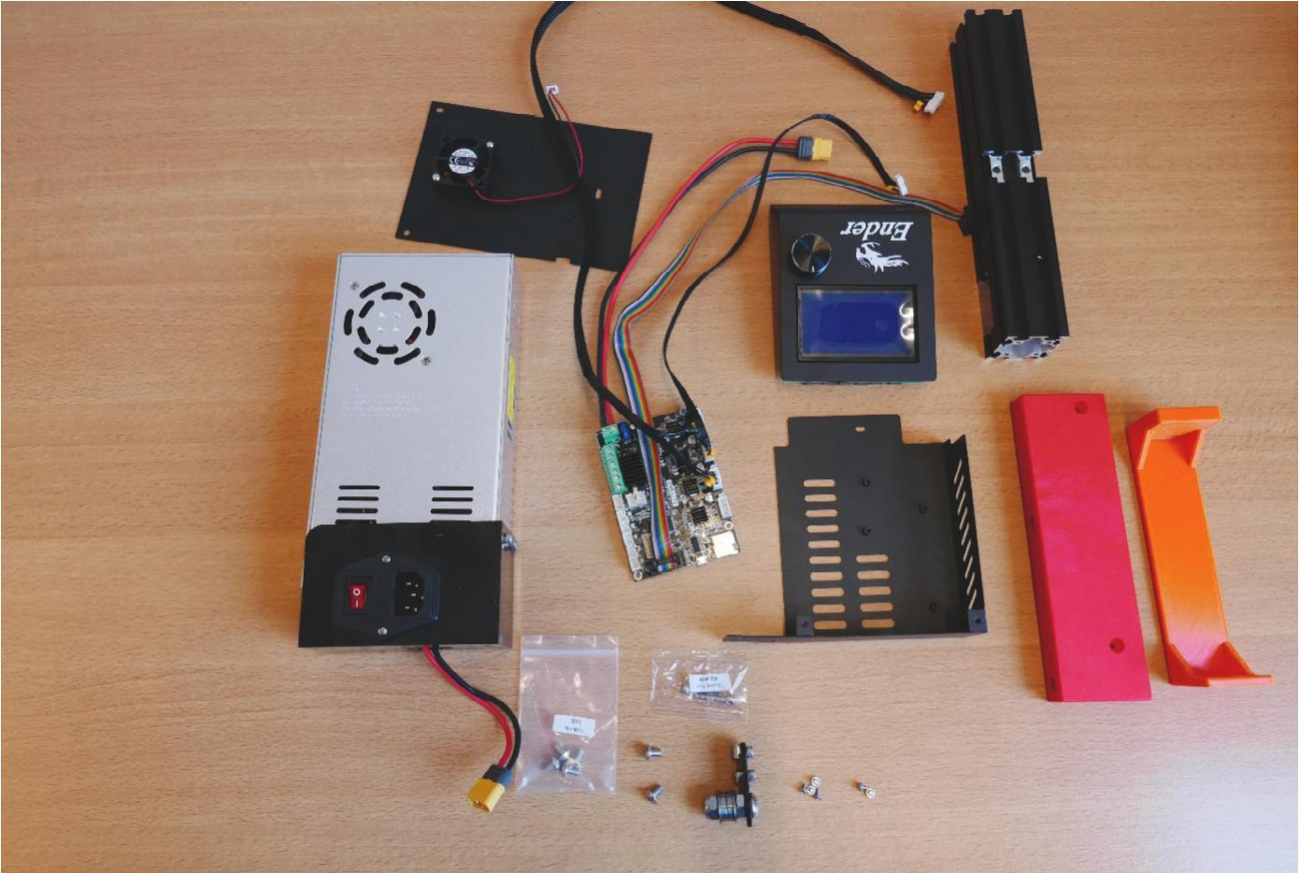
Overview of parts for the Control Unit.

To build the control unit, take the Power Supply Unit (PSU), LCD Screen, 40 × 40 rail, Electronics Box and Board, bag of four M5 × 8 screws, bag of two M4 × 20 screws, X Belt Tensioner, six M3 × 6 screws from the Electronics box, the PSU base 3D print and Electronics Base 3D print (Fig. 36).

**Step 33: Install the LCD on the 40 × 40 rail**

**Fig. 37:**
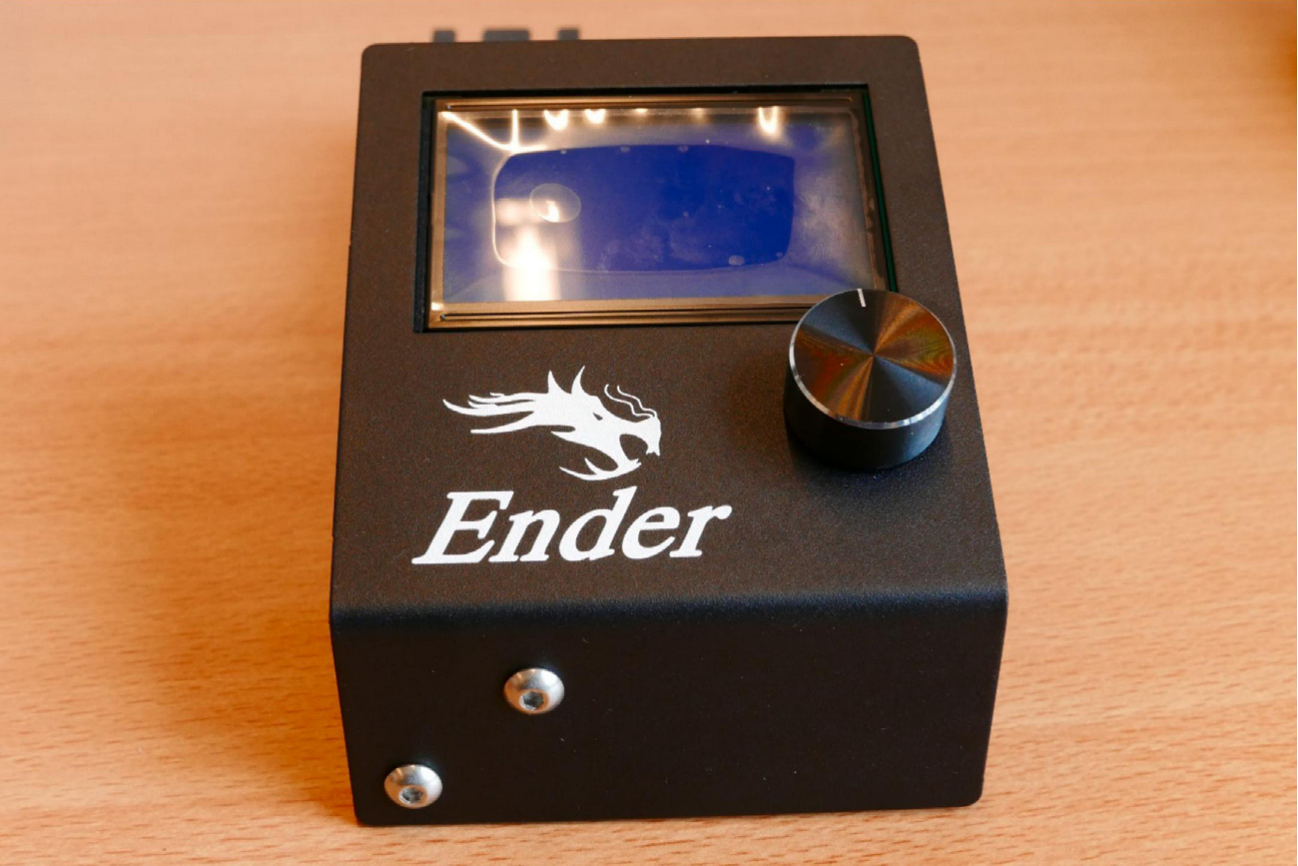
LCD mounted on the 40 × 40 rail.

Using 2 M5 × 8 screws, install the LCD on the 40 × 40 rail (Fig. 37).

**Step 34: Install the PSU on the 40 × 40 rail**

**Fig. 38:**
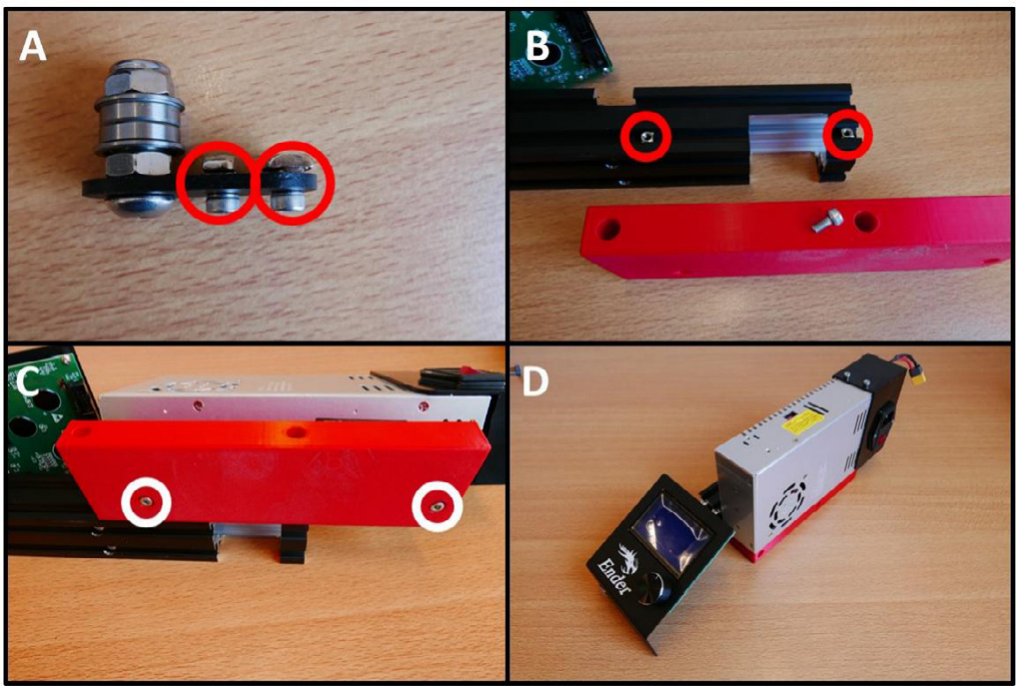
Installing the PSU on the 40 × 40 rail. A) Reuse the M4 screws and T nuts from the X Belt Tensioner. B) Insert T nuts into the rail and screws into the PSU mount. Tighten the screws. C) Screw the PSU to the PSU mount using the M4 × 20 screws. D) PSU mounted to the 40 × 40 rail.

To install the PSU Mount on the 40 × 40 rail, take the M4 screws and M4 T nuts from the X Belt Tensioner (Fig. 38A). Slide the T nuts into the bottom track of the 40 × 40 rail and put the screws into the counterbored holes (Fig. 38B). Tighten the screws to affix the PSU Mount. Next, put the M4 × 20 from the plastic bag into holes to mount the PSU (Fig. 38C). The PSU is now fixed to the rail. Do not open or modify the PSU in any way to prevent the exposure to line voltage. Doing so would risk getting a severe or lethal electric shock.

**Step 35: Reassemble the Electronics Box**

**Fig. 39:**
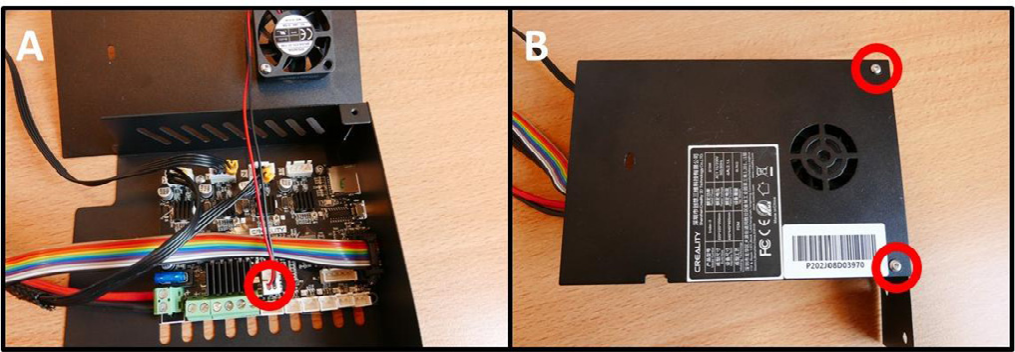
Rebuilding the Electronics Box. A) Electronics Board back in place, fan reattached. B) Cover replaced.

The Electronics Box needs to be reassembled before mounting it on the Control Unit, since it was disassembled for removal of redundant cables. Put the board back in the case using the previously removed M3 × 6 screws. Reattach the fan connector (Fig. 39A). Put the cover back in place with the remaining M3 × 6 screws (Fig. 39B). The (low voltage) electronics are now properly rehoused so the user cannot touch live parts, preventing the risk of electric shock.

**Step 36: Attach the Electronics Base and Electronics Box**

**Fig. 40:**
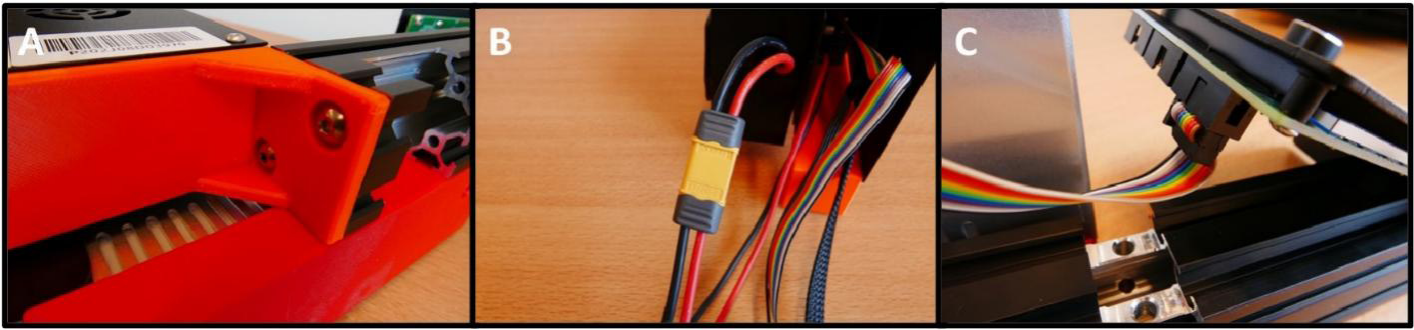
Attaching the Electronics Box to the Control Unit. A) Attach the Electronics Box and Electronics Base. B) Connect to the PSU. C) Attach the rainbow colored cable to the LCD.

To attach the Electronics Box and Electronics Base, sandwich the mounting plates and attach them to the free end of the 40 × 40 rail (Fig. 40A) using two M5 × 8 screws. Attach the Electronics Board to the PSU (Fig. 40B). Attach the rainbow colored cable to the rightmost LCD connector (above the 40 × 40 rail) (Fig. 40C).

**Step 37: Attach the stepper motors to the Control Unit**

**Fig. 41:**
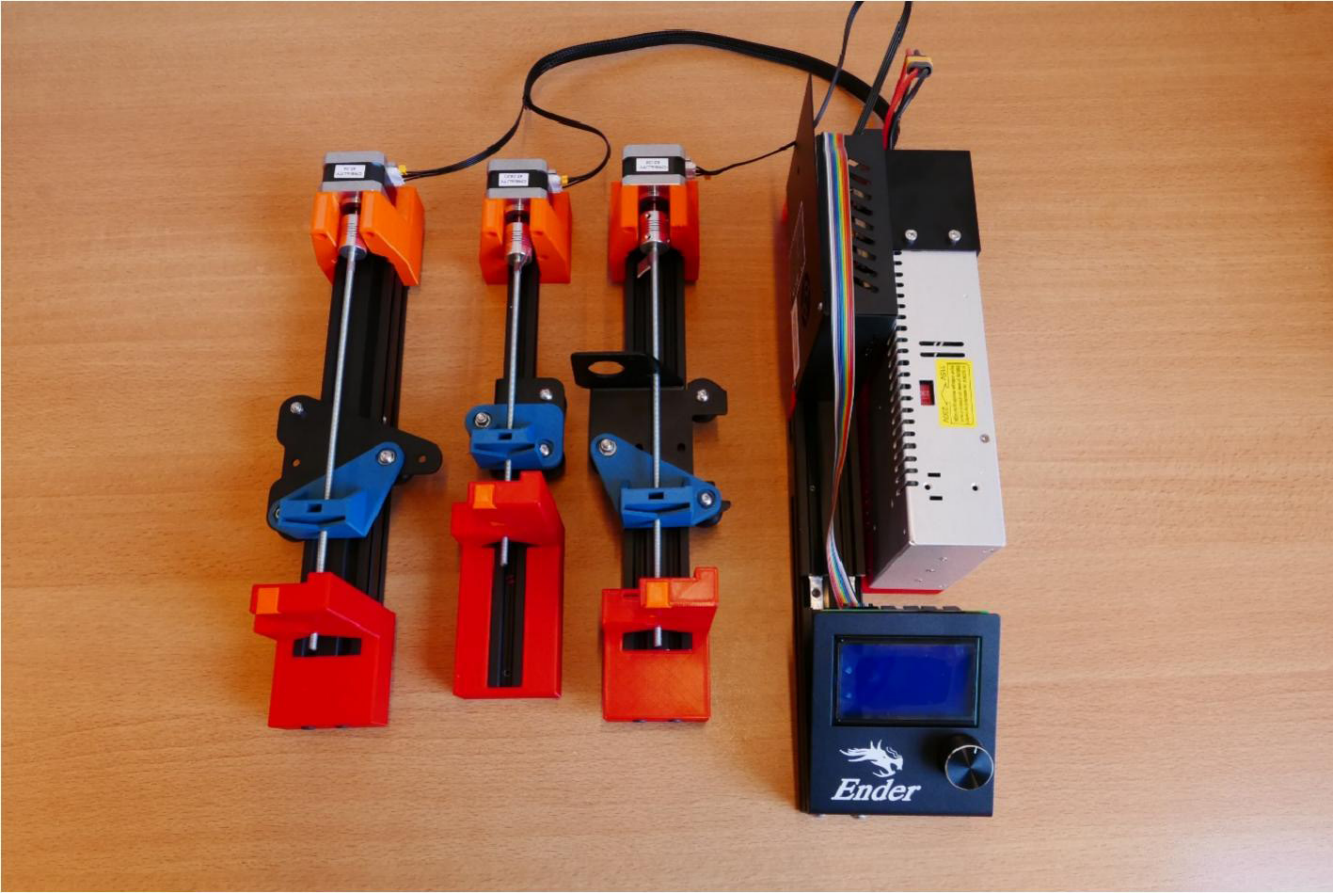
The fully assembled pump system, with the stepper motor cables attached.

The final step in the assembly process is to connect the stepper motor cables to the stepper motors themselves. The final result is shown in Fig. 41. The pump system is now completely built and ready to use.

## Operation instructions

The syringe pumps are controlled by the 3D printer motherboard, which is already flashed with Marlin software and therefore works with G-code. There are two ways of controlling the pumps: a) using a computer connected to the board via USB and using direct control software such as PronterFace [38] or Octoprint [39], with the latter able to be used remotely via network. B) Writing the G-code file, uploading it on a microSD card and using the printer interface for printing the file without the need of a computer attached to the pumps. For more experienced users, a Python based grbl interface can be used to program the pumps [40]. Grbl is another G-code based motion control firmware, from which Marlin was originally derived. Grbl has also been shown to be useful in Open Hardware motion control [41]. Please note that when using these pumps, moving parts are involved, which may pose a pinching hazard. This is similar to commercial syringe pumps, but users need to be aware of potential risks.

A G-code file is a set of instruction which Marlin interprets line by line and passes the operations to the motors. The file is a simple text file saved as .gcode. For controlling the pumps, a few G-code commands are needed:

M92 X <Steps per mm> Y <Steps per mm> Z <Steps per mm>

**M92** should be at the start of the G-code, as this command will set the number of steps required for moving one axis one millimeter, which in the case of the Ender 3 motors/drivers and the M5 × 0.8 mm threaded rod is 4000 steps per mm. The Ender 3 uses stepper motors with a step angle of 1.8°, or 200 steps per revolution. The TSMC2208 stepper drivers are set to 1/16 microsteps, which is the default setting for the Ender 3. The pitch of the leadscrew is 0.8 mm. This gives the value of 4000 steps per mm. However, as we are dealing with flows, we found that it is more user-friendly to change this command from steps per mm to steps per mL. This can be easily done by calculating the distance required to push a volume of 1 mL depending on the diameter of the syringe used. To do this, the inner diameter (I.D.) of the syringe needs to be determined. Next, the area of the plunger can be calculated. The linear syringe travel to deposit 1 mL can be calculated by dividing 1 cm^3^ by the plunger area (cm^2^). Divide this number by the steps per mm value (4000) to get the steps per mL. We have attached a simple calculator in the supplementary material to calculate the steps per mL using the diameter of a syringe as input. As an example, a 10 mL syringe has a diameter of 15.6 mm and the calculated steps required to move 1 mL are 20927, a 5 mL syringe has a diameter of 12.3 mm and the calculated steps are 33663.

Another thing to take note of, is that in the normal configuration, the X and Y motors are inverted, so this should also be set in the M92 command by giving negative step/mm values.

A M92 set up using two 5 mL syringes on the X and Z axes and a 10 mL syringe on the Y is therefore:

M92 X-33663 Y-20927 Z33663

This will set the number of steps per mL in such a way that the rest of the G-code will use this to calculate the pump movements.

**M302 S0**

This command will set the printer to move the motors without checking the temperature of the hot end. This is usually a 3D printer safety check for avoiding extruding material when there are some problems with the hot end. In this case the hot end is detached, so there is no risk of thermal runaway.

**M211 S0**

This command will disable the endstops which may cause problems as they are not connected.

**G91**

Use relative positioning. This is important when multiple movements are used, otherwise the motors will start from a 0.0.0 position and use that as reference point.

**G4 S <time in seconds>**

Pause the commands for a set amount of time.

**G1 X <mL> Y <mL> Z <mL> F <mL/min>**

This is the command to move the syringe pumps at certain flowrates. As we set up the steps/mL in the M92 command, now all the G1 codes are in mL/min. This can be a positive number or a negative number, positive for pushing and negative for retracting.

For example:

G1 X0.5 F1

Will use syringe pump X and will deliver 0.5 mL of liquid at a speed of 1 mL per minute.

Multiple syringes can be used at the same time:

G1 X0.5 Y0.5 Z0.5 F1

Will deliver, at the same time, 0.5 mL of liquid from the pumps on X, Y, and Z at a speed of 1 mL/min

For using different flow speed for different pumps, the total amount of liquid should be calculated taking in account that all the pumps will reach the end at the same time. For example:

G1 X2 Y1 Z2 F1

Will deliver the set amount of liquid at different flow speed, using 1 mL/min on Y, and 2 mL/min on X and Z.

For commenting on G-code files the “;” character should be used. This can be useful in commenting the G-code with metadata.

An example of full G-code:

(Video S1, using 1 mm OD, 0.8 mm ID silicon tubing, press-fit in the PDMS device)

*; Experiment 01 20/05/2021*

*; 10 mL syringes on the three pumps*

M92 X-20927 Y-20927 Z20927; set the proper steps/mL for the 10 mL syringe

M302 S0; print without checking temperature

G91; relative positioning

*; deliver 1 mL at 2 mL/min sequentially*

G1 X1 F2

G1 Y1 F2

G1 Z1 F2

*; wait for 10 s*

G4 S10

*; deliver 1 mL on X and Z at 1 mL/min, 2 mL on Y at 2 mL/min*

G1 X1 Y2 Z1 F1

*; wait 10 s*

G4 S10

*; deliver 1 mL at 2 mL/min sequentially*

G1 Z1 F2

G1 Y1 F2

G1 X1 F2

Without previous knowledge in programming and in a few lines of G-code it is possible to program the syringe pumps to do multiple operations. This file can now be saved as .gcode, uploaded on a microSD card, and run on the Ender 3 syringe pumps. Or it can be sent using a computer connected to the USB.

G-code files can be easily shared between labs for improving reproducibility of experiments.

## Validation and characterization

Pumps using designs for translating rotational motion to linear motion using a lead screw, are affected by the quality of the motors/drivers and by the structural stability of the apparatus. The motors and drivers used here have been used in 3D printing since many years, and usually a failed 3D print can be traced back to a failure in the hot-end or the extruder, and only rarely to the XYZ motion of the printer.

The linear rail and the bearings system are also directly translated from the 3D printing motion to the syringe pump motion. And in this case, if the wheel pressure is properly calibrated to the extruded aluminum frame, there are no possible failures.

As shown in the build instruction, we screw all the 3D printed parts on the frame and checked that there was no loose movement.

With this set-up we managed to reproducibly deliver 10 µL of water using a 1 mL syringe pump. We repeated the command 10 times, using an analytical balance to weight the delivered liquid. We measured an average of 10.4 mg with an error of ±0.3 mg, which is indistinguishable from the error of the analytical balance used.

The amount of delivered liquid, as explained in the previous section, is dependent on the steps per mL, and this can be finely tuned using an analytical balance and changing the values in the command M92.

Another important thing is not only the amount of liquid delivered, but the flow stability. This is especially interesting in microfluidics, where the flow at low speed, should be as stable as possible.

We show how the flow from two or three pumps is stable in at low flow speed and in microfluidic devices. In Video S2, we used two 5 mL syringes for delivering 50 µL at a speed of 5 µL/min in a PDMS/Glass microfluidic Y-channel 2 mm wide and with a height of 0.5 mm. Even using such a large syringe, the laminar flow between the two flows is barely disturbed during the experiment.

Similarly, we used three 10 mL syringes for flowing 50 µL at 25 µL/min in a three-way-in single PDMS microfluidic channel 1 mm wide and 0.25 mm height. Also in this case the three flows are barely disturbed at their interfaces.

We also succeeded in forming a multiphase system using the same three way in microfluidic device, where two inlets were used for flowing oil, and the central channel was used for the water phase (Video S3). In this case, we used three syringe pumps delivering 50 µL at a speed of 25 µL/min.

## Declaration of Competing Interest

The authors declare that they have no known competing financial interests or personal relationships that could have appeared to influence the work reported in this paper.
